# The RyfA small RNA regulates oxidative and osmotic stress responses and virulence in uropathogenic *Escherichia coli*

**DOI:** 10.1371/journal.ppat.1009617

**Published:** 2021-05-27

**Authors:** Hicham Bessaiah, Pravil Pokharel, Hamza Loucif, Merve Kulbay, Charles Sasseville, Hajer Habouria, Sébastien Houle, Jacques Bernier, Éric Massé, Julien Van Grevenynghe, Charles M. Dozois

**Affiliations:** 1 INRS-Centre Armand-Frappier Santé Biotechnologie, Laval, Québec, Canada; 2 CRIPA-Centre de recherche en infectiologie porcine et avicole, Saint-Hyacinthe, Québec, Canada; 3 Department of Biochemistry, RNA Group, Université de Sherbrooke, Sherbrooke, Quebec, Canada; INSERM U1220, FRANCE

## Abstract

Urinary tract infections (UTIs) are a common bacterial infectious disease in humans, and strains of uropathogenic *Escherichia coli* (UPEC) are the most frequent cause of UTIs. During infection, UPEC must cope with a variety of stressful conditions in the urinary tract. Here, we demonstrate that the small RNA (sRNA) RyfA of UPEC strains is required for resistance to oxidative and osmotic stresses. Transcriptomic analysis of the *ryfA* mutant showed changes in expression of genes associated with general stress responses, metabolism, biofilm formation and genes coding for cell surface proteins. Inactivation of *ryfA* in UPEC strain CFT073 decreased urinary tract colonization in mice and the *ryfA* mutant also had reduced production of type 1 and P fimbriae (pili), adhesins which are known to be important for UTI. Furthermore, loss of *ryfA* also reduced UPEC survival in human macrophages. Thus, *ryfA* plays a key regulatory role in UPEC adaptation to stress, which contributes to UTI and survival in macrophages.

## Author summary

Small regulatory RNAs play a critical role in bacterial adaptation, physiology, and metabolism. We investigated the role of the small RNA *ryfA* in uropathogenic *E*. *coli*, by using genetic, molecular and cell culture approaches, and mouse infection models. We demon-strate that RyfA is important for resistance to oxidative and osmotic stresses and affects the global transcription of multiple responses to stress. Deletion of *ryfA* also reduced production of type 1 and P fimbriae (pili) and increased swimming motility. RyfA also plays an important role in urinary tract infection (UTI) in the mouse model and for survival in human primary macrophages and cell lines. Elucidating a role for RyfA in adaptation to stress response and virulence provides insight into new regulatory mechanisms important for virulence in pathogenic *E*. *coli* and likely other Enterobacteria that may lead to development of targeted therapeutic or prophylactic approaches against these pathogens.

## Introduction

Urinary tract infections (UTIs) are one of the most prevalent bacterial infections, affecting millions of people each year [1]. UTIs primarily affect women, and up to 50% of adult women have experienced at least one UTI episode during their lifetime. Recurrent UTIs are observed in a quarter of women within 6 months of initial diagnosis and in half of the women within one year of a UTI episode even after antimicrobial treatment [2]. Uropathogenic *Escherichia coli* (UPEC) remains, by far, the primary causative agent of uncomplicated UTIs [1]. To establish, maintain, and to circumvent host defenses during an infection, UPEC are equipped with specialized virulence factors. Well-documented examples include adhesins (type 1, P, F1C, and S fimbriae), flagella, iron acquisition systems, a polysaccharide capsule and toxins such as hemolysin [3,4].

Bacterial adherence to host cells is critical for initiating pathogenesis and persistence during a UTI. Fimbrial adhesins (type 1, P, F1C, S, and Dr) and nonfimbrial adhesins (TosA) mediate binding to cells lining the urinary tract mucosal surfaces [5,6]. Type 1 fimbriae encoded by the *fimAICDFGH (fim)* operon are critical for colonization and invasion of bladder epithelial cells. They aid in formation of biofilm-like intracellular bacterial communities (IBCs) that contain large numbers of bacteria which may persist as reservoirs and may lead to recurring infections [7]. UPEC type 1 fimbriae bind to mannosylated uroplakin proteins enriched on the apical surface of the bladder [8]. *fimA* encodes the major subunit of type 1 fimbriae and *fimH* encodes the mannose-specific adhesin. The promoter region regulating *fim* expression (*fimS*) is located on a 314-bp invertible element flanked by two 9-bp inverted repeats, and the orientation of *fimS* provides a mechanism of phase variation wherein a bacterial cell produces type 1 fimbriae, *fimS* in the ON orientation, or does not produce, *fimS* in the OFF orientation [9]. FimB and FimE are site-specific recombinases involved in phase variation (flipping) of *fimS*. FimB mediates the ON-to-OFF or OFF-to-ON inversions of *fimS*, and FimE preferentially mediates the ON-to-OFF inversion of *fimS* [10]. In addition, in UPEC strain CFT073 there are three additional tyrosine recombinases, IpbA, IpuA, and IpuB, which are also capable of catalyzing inversion of *fimS* [11]. Numerous mechanisms of regulation of the *fimS* switch and expression of type 1 fimbriae have been described. Regulatory proteins such as integration host factor (IHF), leucine responsive protein (LRP), and histone-like nucleoid structuring (H-NS) affect *fim* expression [12]. Other factors have also been shown to affect type 1 fimbriae expression, including cross-talk with genes from other fimbriae [13] and environmental conditions such as pH, osmolarity, temperature, metabolite availability and oxygen levels [14,15].

As with all infectious agents, UPEC must circumvent, suppress, or resist a variety of stressful conditions and host defenses during the course of an infection. In the urinary tract, UPEC may encounter neutrophils and macrophages which produce antimicrobial factors, such as reactive oxygen species (ROS) and reactive nitrogen species (RNS) [16]. ROS and RNS can cause damage and physiological stress with pleiotropic effects on bacterial cells. These products can damage the bacterial membrane and DNA, alter enzyme activity by damaging iron-sulfur clusters in enzymes and cause oxidation of proteins and lipids [17,18]. Indeed, UPEC need to overcome environmental stresses that can be encountered during infection by a variety of mechanisms, and it is thought that resistance to ROS may be important for UPEC pathogenesis.

Bacterial small RNAs (sRNAs) are known regulators of many physiological processes [19]. Several sRNAs play key roles in bacterial adaptation to changing environmental conditions and stress responses, including responses to nutrient availability, envelope and osmotic stresses, oxidative stress, iron deficiency, and pH stress [20–22]. *ryfA* encodes a sRNA [23] and had been implicated in biofilm formation *[24,25]* and swarming motility [25] in *E*. *coli* and pathogenesis in *Shigella dysenteriae [26].* A toxic peptide TimP (for toxic inner membrane protein), identified when the *ryfA* paralog in *Salmonella enterica* was highly expressed, was recently reported [27].

The purpose of this research was to investigate the role of the sRNA RyfA for gene regulation in UPEC and its importance for adaptation to stress and colonization during UTI. Here, we show that RyfA contributes to resistance to oxidative and osmotic stresses in UPEC strain CFT073. In both *in vitro* and *in vivo* experiments, deletion of *ryfA* resulted in important differences in expression of genes that contribute to protection against environmental stresses, and that are known to regulate fimbrial adhesins, cellular processes, and metabolism. These regulatory changes are likely to contribute to attenuation of UPEC and its decreased capacity to survive within macrophages. These data support the hypothesis that RyfA is involved in resistance to oxidative stress in UPEC and that RyfA can also influence type 1 fimbriae expression, thereby attenuating UPEC virulence. Understanding the link between the RyfA small RNA and its connection between stress responses, and expression of type 1 fimbriae will better elucidate the signals that control UPEC virulence, and may lead to the development of therapeutics that could potentially inhibit the establishment of UTI and thereby reduce the occurrence of this highly prevalent infection.

## Results

### The *ryfA* gene is involved in resistance to osmotic stress

**Fig 1:**
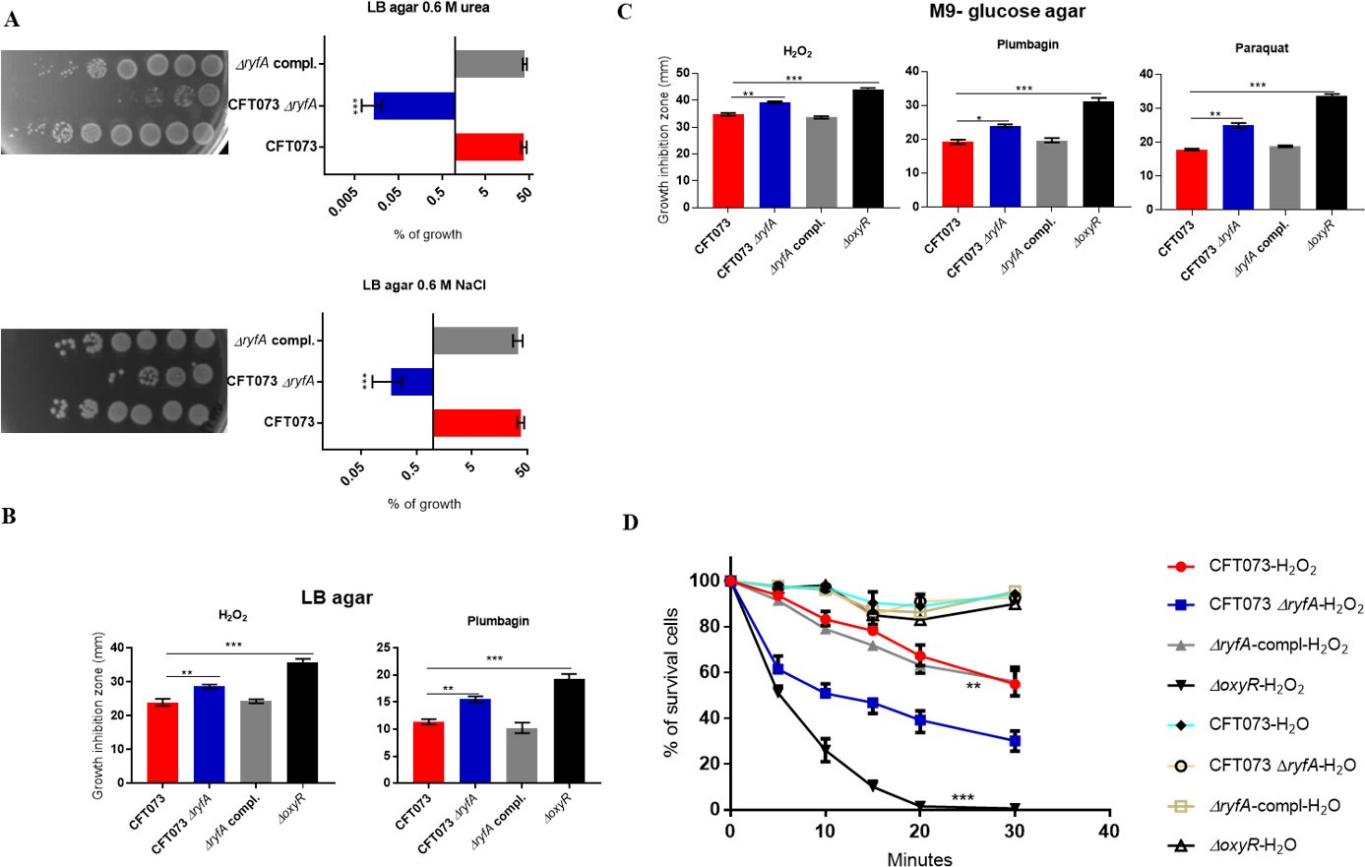
Role of RyfA in osmotic and oxidative stress resistance. (**A**) Growth under conditions of osmotic stress. Strains were grown shaking in LB medium until mid-log phase (O.D._600_, 0.6) and plated on LB agar and LB agar with 0.6 M NaCl and 0.6 M urea (see Methods). (**B**) Growth inhibition zones (mm) of UPEC CFT073, *ryfA* mutant and complemented strains to oxidative stress generating compounds. Strains were grown on either LB agar or (**C**) M9- glucose agar. ROI-generating compounds tested were 30% hydrogen peroxide (H_2_O_2_), 53 mM plumbagin, or 40 mM paraquat. (**D**) Resistance to hydrogen peroxide in LB broth. WT CFT073 and *ryfA* mutant strains were examined in LB medium containing 5mM H_2_O_2_ or an equivalent volume of Milli-Q water (H_2_O). Samples were collected every 5 minutes, diluted, and plated on LB agar to determine CFUs. The CFT073 Δ*oxyR* strain was used as a sensitive control strain. The results represent the means of replicate experiments for a minimum of three samples. Vertical bars represent the standard errors of the means. Statistical significance was calculated by one-way ANOVA (A to D): (*, *P* < 0.05; **, *P* < 0.005; ***, *P* < 0.0001).

We previously generated a transposon (Tn) library in UPEC reference strain CFT073 [28]. The library was subjected to osmotic and oxidative stresses as a selection for potentially decreased fitness in an environment in which the bacteria encounter host-derived stresses including osmolarity in urine and interactions with host immune cells. We screened this Tn library for mutants that exhibited reduced tolerance to osmotic and oxidative stress in order to identify genes that may play a role in UPEC stress tolerance. One mutant that exhibited reduced tolerance to osmotic and oxidative stress carried a transposon insertion adjacent to the *ryfA* gene. To confirm the direct role of *ryfA* in phenotypes of decreased tolerance to stress, we generated a site-directed deletion to create a Δ*ryfA* mutant of UPEC strain CFT073. To determine whether *ryfA* contributes to resistance to osmotic stress, the WT parental strain, Δ*ryfA*, and a complemented Δ*ryfA* strain were subjected independently to osmotic stress (0.6 M Urea) and salt stress (0.6 M NaCl), on modified LB agar. The WT and the complemented mutant were able to grow on LB agar containing NaCl or urea (Fig 1A). By contrast, on LB agar with 0.6 M urea or 0.6 M NaCl, the capacity of the *ryfA* mutant to grow was significantly reduced (Fig 1A). In LB agar, there was no growth difference between CFT073 and CFT073 *ΔryfA* (S1A Fig). This result suggests that RyfA could play a role in resistance to osmotic stress caused by NaCl and urea.

### Loss of *ryfA* increases sensitivity to ROI-generating compounds

To determine if *ryfA* influences UPEC resistance to oxidative stress, bacterial survival after exposure to oxidative stress was tested using different reactive oxygen intermediate (ROI)-generating agents. We used H_2_O_2_ and various superoxide generators such as plumbagin and paraquat, during growth on either rich (LB) or minimal (M9-glucose) medium. Sensitivity to these compounds was determined with wild-type UPEC strains CFT073 and 536 and corresponding Δ*ryfA* mutants. On LB medium, the CFT073 Δ*ryfA* strain was more sensitive to the ROI-generating compounds H_2_O_2_ (p = 0.0022) and plumbagin (p<0.0001) when compared to parental strain CFT073 (Fig 1B). The UPEC 536 Δ*ryfA* mutant was only more sensitive to H_2_O_2_ (p = 0.0065) when compared to strain 536 (S1B Fig). On minimal medium, both UPEC *ryfA* mutants were more sensitive to H_2_O_2_, plumbagin, and paraquat (Figs 1C and S1B). In addition, in LB broth the CFT073 Δ*ryfA* mutant was killed more readily compared to wild-type CFT073 (p = 0.0057) after exposure to 5 mM hydrogen peroxide (Fig 1D). Complementation of the Δ*ryfA* mutant with a single-copy of *ryfA* on the chromosome restored resistance to ROI-generating compounds (Figs 1B, 1C and 1D). These results show that loss of the *ryfA* sRNA reduces bacterial resistance to oxidative stress, and that RyfA may contribute to regulation of genes implicated in the oxidative stress response.

### RyfA is produced in logarithmic phase

*ryfA* was originally identified by *in silico-*based genomic analyses designed to identify genes encoding previously uncharacterized sRNA molecules [23]. *In silico* analyses revealed that *ryfA* is chromosomally located between *sseA* and *sseB* (S2A Fig). Using BLAST searches, we identified *ryfA* homologues in other species of *Escherichia*, *Salmonella*, *Shigella*, *Klebsiella*, *Enterobacter*, *Serratia and Citrobacter* (S2A Fig). *ryfA* from CFT073 shares 98% identity at the nucleic acid level with the non-pathogenic K-12 strain MG1655 (S2B Fig), and has greater than 90% sequence identity to *ryfA* sequences from other intestinal and extraintestinal pathogenic *E*. *coli* and *Shigella* spp. (S2A Fig).

**Fig 2:**
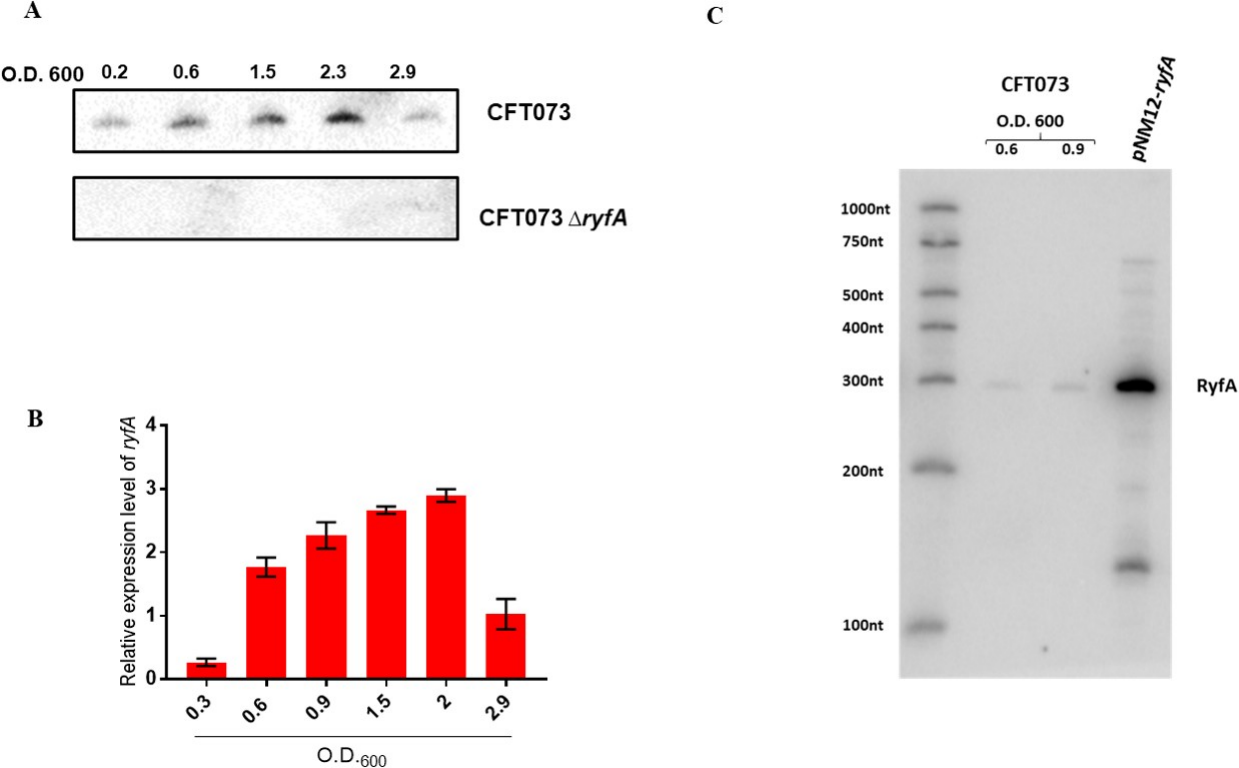
CFT073 produces RyfA in mid-logarithmic phase. (**A**) Northern blot analysis using 10 mg of total RNA isolated from WT CFT073 following growth in LB medium at 37°C. Lack of signal in Δ*ryfA* strain confirms absence of RyfA in the Δ*ryfA* strain. (**B**) Quantitative real-time PCR (qRT-PCR) analysis demonstrating that RyfA is produced by wild-type CFT073 under the conditions tested. (**C**) Northern blot showing RyfA expression in a strain overexpressing RyfA.

To determine under what conditions CFT073 produces RyfA, we performed Northern blotting under laboratory conditions. Bacteria were grown in LB medium at 37°C until the culture O.D. of 0.2 to 2.9. Northern blot analysis showed that RyfA was expressed starting at mid-log phase and reached maximal production in late exponential phase (~ 0.9 to 2.3) (Fig 2A). By using quantitative real-time PCR (qRT-PCR) analysis we confirmed that RyfA is produced by wild-type CFT073 under the same conditions (Fig 2B). We did not detect any signal in the Δ*ryfA* strain, confirming the absence of RyfA in the mutant strain (Fig 2A).

*ryfA* is predicted to be 305 nt in length (S2B Fig). The expression of *ryfA* was placed under the control of an arabinose inducible promoter and the size of the *ryfA* transcript was confirmed by radiolabeled *ryfA*-specific antisense RNA probe. A single band of approximately 300 nt was detected by northern blot in RNA samples obtained from strain CFT073 and using the inducible promoter construct (Fig 2C). The secondary structure of RyfA was predicted using the Vienna RNA websuite program [29], and its predicted secondary structure is comprised of multi-stem loops (S2C Fig).

### Transcriptomic analysis of the effect of loss of *ryfA* on UPEC gene expression

**Fig 3:**
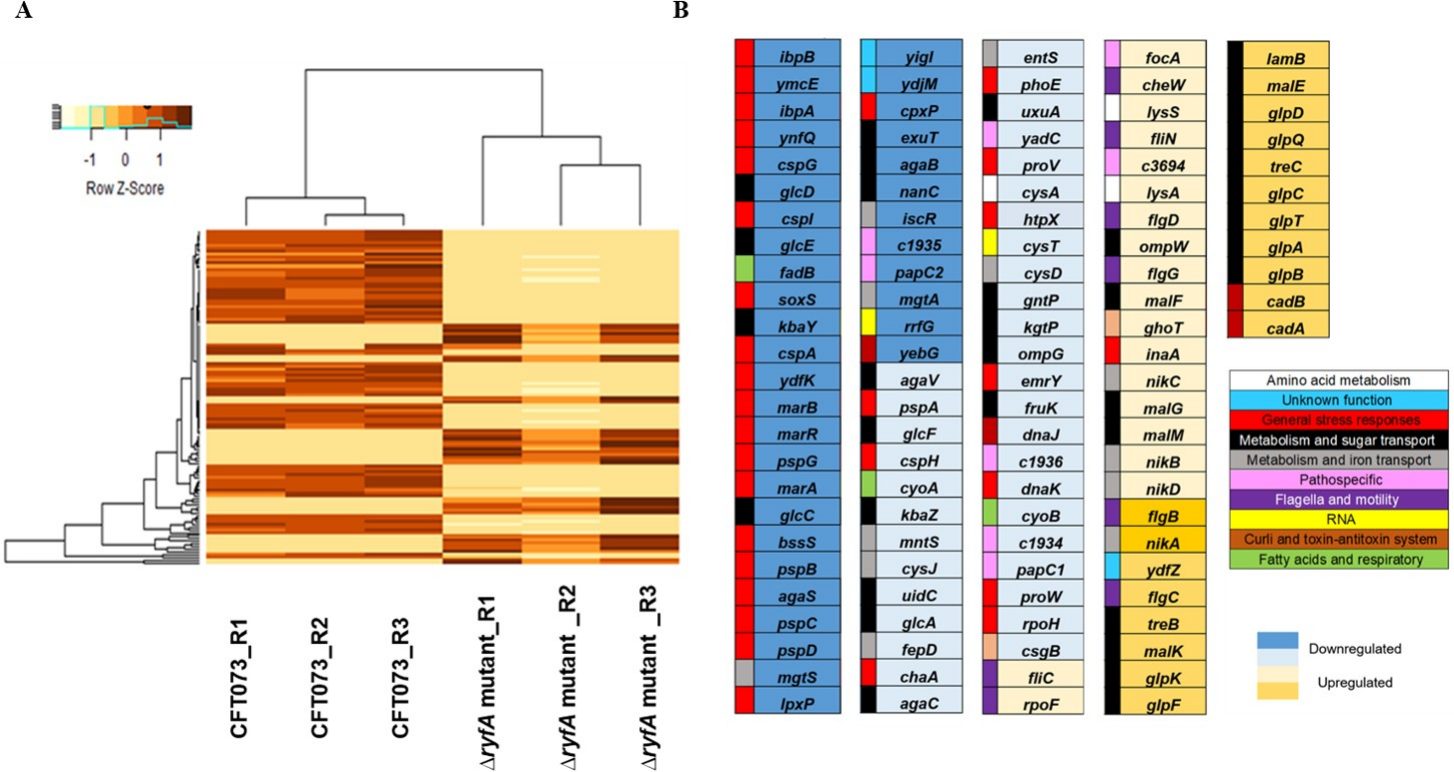
Genes significantly affected by deletion of *ryfA*. RNA-seq analysis was performed on RNA samples (in triplicate) from strain CFT073 and CFT073 Δ*ryfA* grown in LB at mid-log phase of growth (O.D. 0.6). (**A**) Heatmap of the entire data set (n  =  6) of CFT073 and *ΔryfA* mutant. Each row of the heatmap represents the log2 fold values transformed with Z-score of 121 differentially expressed genes (upregulation, red, downregulation, yellow). Hierarchical grouping of differentially expressed genes shows clustering. Z-scores are computed on a gene-by-gene basis by subtracting the mean and then dividing by the standard deviation. Specific genes and changes are presented in Fig 3B. (**B**) Genes classified in different categories based on log2 fold-change difference. Genes upregulated (yellow rectangles) and downregulated (blue rectangles) by at least 1.7-fold were considered significant with P < 0.05. Dark blue is greater or equal to (≥) a 3-fold decrease. Light blue is a decrease from 3-fold to 1.7-fold. Light yellow is an increase from 1.7-fold to 3-fold. Dark yellow is a greater or equal to (≥) 3-fold increase. Genes are classified in different categories with colored squares: General stress responses in red, metabolism and sugar transport in black, metabolism and iron transport in grey, patho-specific in pink, flagella and motility in purple, fatty acids and respiratory chain in green, amino acid metabolism in white, curli and toxin-antitoxin system in orange, RNA in yellow and unknown function in azure. Specific values fold-changes are presented in S1 Table (see supplementary data).

Our *in vitro* experiments revealed an important role for RyfA in response to stresses. We reasoned that the greater sensitivity of the *ryfA* mutant could be due to numerous changes in gene expression that occur in the absence of this regulatory RNA. To examine the role of RyfA, we performed RNA-Seq to compare RNA profiles between wild-type *E*. *coli* CFT073 and the CFT073 Δ*ryfA* mutant. Bacteria were cultured in triplicate at 37°C in LB with aeration to mid-logarithmic growth (O.D. 0.6), mRNAs were extracted and RNA-Seq was performed. In total, the deletion of *ryfA* significantly altered the expression of 484 genes (120 were upregulated and 364 were downregulated) with a log2 fold-change (FC) greater than or equal to ± 1.7 (S1 Table). The genes that were most highly affected in response to loss of *ryfA* are listed in Fig 3. Functional analysis revealed that the genes belonging to the categories “metabolism” and “stress response” were by far the most markedly affected. Indeed, increased sensitivity to stresses is likely a consequence of altered gene expression and defective regulation of the stress response.

#### Loss of *ryfA* alters expression of genes regulated by the RpoH (σ^32^) (heat shock response) regulon

*rpoH* encodes σ^32^, the primary sigma factor controlling the heat shock response during log-phase growth. It is subjected to tight control via a multivalent regulatory system that responds to temperature and the abundance of misfolded proteins within the cell [30]. During exponential growth, in the *ryfA* mutant, *rpoH* gene expression was down-regulated (-1.76-fold) (Fig 3). The heat shock regulon mediated by σ^32^ controls a major stress response to cope with heat and other stresses in *Escherichia coli* [31]. The heat-shock response is a protective mechanism that is crucial for bacterial survival and adaptation to hostile environmental conditions. The most down-regulated genes in the *ryfA* mutant were genes encoding both cold shock and heat shock proteins (Fig 3B). These included genes encoding chaperones involved in protein fate, such as those responding to heat shock: *ibpB* (-7.70-fold), *ibpA* (-5.96-fold), *dnaKJ* (-2.20-fold), *htpX* (-2.28-fold) and *cpxP* (-3.37-fold). CpxP functions as both a chaperone and a repressor of the Cpx response. This system is involved in many cytoplasmic events, such as folding of nascent polypeptide chains, rescue of misfolded proteins and assembly and disassembly of protein complexes [32,33].

#### Alteration of expression of genes involved in the general stress response

Several genes that are involved in defense against oxidative stress were also down-regulated including *soxS*, which encodes the transcriptional regulator that responds to superoxide-generating species (-5.03-fold) (Fig 3B and S1 Table). The four cold shock genes *cspG*, *cspI*, *cspA cspH* were also down-regulated. Other genes associated with cold-shock were downregulated such as *ymcE* (-6.39-fold) and *ydfK* (-4.07-fold), as well as genes in the *pspABCDG* operon coding for phage shock proteins. The Psp system protects proteins from aggregation and helps maintain the proton motive force (PMF) to counteract stress conditions (such as filamentous phage infection, extreme temperatures, osmolarity changes, and the mislocalization of secretin proteins) [34]. The genes *yebG* and *ydjM*, which are induced during stress involving DNA-damage and the SOS response were also downregulated. In addition, *emrY* (-2.22-fold), encoding a multidrug resistance secretion protein and also known to reduce the lethal effects of stress was downregulated (S1 Table).

Further, other genes that contribute to resistance to antimicrobials, toxic compounds and oxidative stress were also downregulated in the *ryfA* mutant. These include the *marR* (-4.01-fold) gene encoding the multiple antibiotic resistance regulator and *marAB* (-3.92- to -4.01-fold) encoding multiple antibiotic resistance proteins. Interestingly, *proVW*, encoding systems for the transport of the osmoprotectants proline and glycine betaine were also downregulated (-1.94 and -2.29-fold, respectively) (Fig 3B). The downregulation of stress response genes corresponds to the increased sensitivity to oxidative and osmotic stress that was determined (Fig 1).

#### Loss of *ryfA* altered expression of genes encoding fimbriae and required for flagella synthesis and motility

RNA-Seq results indicated that deletion of *ryfA* altered expression of genes encoding different types of fimbriae. We observed significant downregulation of the *papC* genes, which encode the usher protein required for biogenesis of P fimbriae. The CFT073 genome harbors two copies of the *pap* operon, designated *pap1* and *pap2*, and both *papC1* and *papC2* genes were repressed (-2.02 and -3.16-fold, respectively) (Fig 3B and S1 Table). The *f9* genes, encoding F9 fimbriae (ORFs *c1931*-*c1936*), showed decreased expression (-3.18 to -1.61-fold). The *yadC* gene (*c0166*), encoding a putative fimbrial adhesin protein, was also down-regulated (-2.3-fold). In contrast, *focA* (c1239) encoding the F1C fimbrial major subunit protein was increased (1.81-fold) (Fig 3B and S1 Table). While we identified some differentially regulated fimbria-encoding genes within the *yeh*, *yqi*, and *yfc* operons, most genes within these operons were either not differentially regulated or were excluded due to having mapped reads below the cutoff value.

The gene encoding the sigma factor 28 (σ^28^), which is responsible for initiation of transcription of a number of genes involved in motility and flagellar synthesis was up-regulated (1.73-fold). In agreement with this, some flagellar biosynthesis and motility genes were also up-regulated (*flgCBGD*) (2.18 to 3.48-fold), the *fliC* gene encoding flagellin (1.60-fold), and *cheW* (1.90-fold), which encodes a positive regulator of chemotaxis (Fig 3 and S1 Table).

Some genes that are involved in biofilm formation were also downregulated, including, genes important for cellulose synthesis (*bssS)* (-3.79-fold), thin aggregative curli fiber production (*csgB*) (-1,76-fold) and lipid A biosynthesis (*lpxP*) (-3,51-fold) (Fig 3B). We were unable to determine if *csgA*, *csgD*, *csgE*, and *csgF* were differentially expressed, as their expression levels were too low under the test conditions that were used and were excluded from the final RNA-Seq analysis. Taken together, deletion of *ryfA* altered expression of multiple operons encoding fimbriae, downregulated some genes important for biofilm formation and upregulated genes required for flagellar assembly and motility.

### Validation of differentially expressed genes

To validate some of the data obtained from the RNA-Seq results, we performed qRT-PCR using primers specific to a series of genes that were differentially expressed: *soxS*, *ibpA*, *cspA*, *marA*, *bssS*, *rpoH*, *cadA malE* and *treC* to compare log2 fold change in gene expression between CFT073 *ΔryfA* and the CFT073 wild-type strains. Strains were cultured under conditions identical to our RNA-Seq experiment. We observed highly similar differences in gene expression as compared to our RNA-Seq results. Specifically, we observed downregulation of *soxS*, *ibpA*, *cspA*, *marA*, *bssS*, *rpoH* and upregulation of *cadA*, and *treC* (S3A Fig).

### Inactivation of RyfA reduces production of type 1 fimbriae *in vitro*

**Fig 4:**
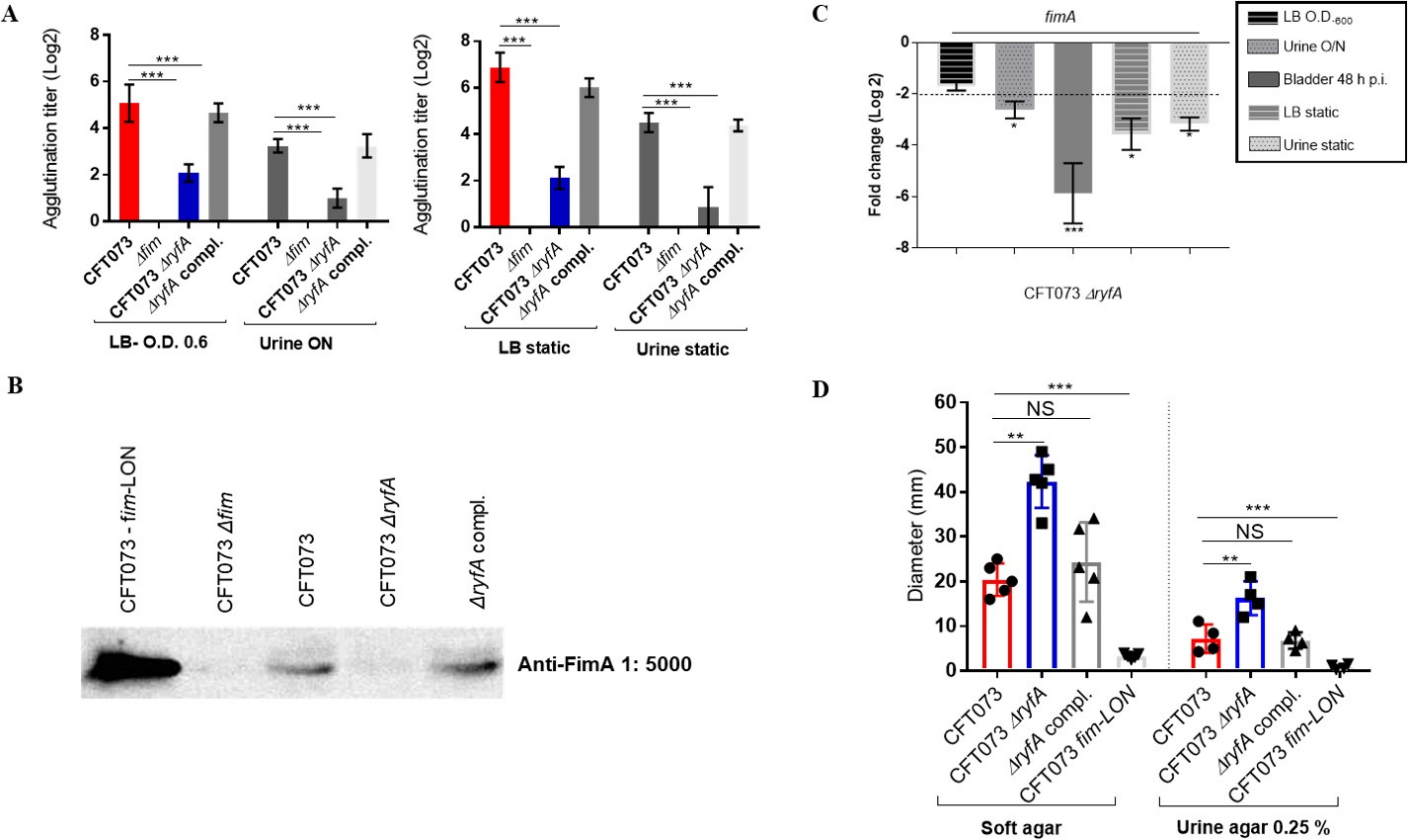
Effect of deletion of *ryfA* on type 1 fimbriae (pili) production and motility. (**A**) Yeast agglutination titer demonstrating level of production of type 1 fimbriae in strains cultured to the mid-log phase and after overnight static growth in LB broth and in human urine. The *Δfim* strain was used as a negative control and showed no agglutination. (**B**) Western blot of fimbrial extracts of strains cultured to the mid-log phase of growth in LB broth. The CFT073 *fim-*locked ON strain was used as a positive control. (**C**) Expression of *fimA* gene by qRT-PCR in the *ΔryfA* strain compared to the WT CFT073 strain in LB, human urine and infected bladders. (**D**) Motility of CFT073, *ryfA* mutant and complemented strain on 0.25% soft or urine agar. Each box and scatter dot plot (min to max) represents the mean diameter of the motility zone. Results are the mean values and standard deviations for at least three biological experiments. Statistical significance was calculated by the Student t test (A, C, D): *, P < 0.05; **, P < 0.005; ***, P < 0.0001. NS, not significant.

Since the *ryfA* mutant is more sensitive to oxidative and osmotic stresses and given that type 1 fimbriae play an important role for colonization in the murine UTI model [5], the effect of the *ryfA* mutation on production of type 1 fimbriae was investigated. Production of type 1 fimbriae was determined by yeast agglutination at the mid-log phase of growth in LB medium (O.D. 0.6) and human urine (O.D. 0.4) and in static conditions. Following mid-log growth with shaking in LB broth, and overnight growth in human urine with shaking, the agglutination titer of CFT073 Δ*ryfA* was significantly reduced (p<0.0001 and p = 0.0062, respectively) as compared to parent strain CFT073 (Fig 4A). When cultured statically, agglutination titers were significantly reduced in the CFT073 Δ*ryfA* mutant compared to the WT strain in LB, human urine (Fig 4A) or minimal M9-glucose broth (S4A Fig). The yeast agglutination titer was also significantly reduced in both UPEC *ΔryfA* strains compared to wild-type UTI89 and 536 parent strains after mid-log phase in LB and human urine (S4B Fig). Yeast agglutination by the *ryfA* complemented mutant regained titers similar to that of the WT strain (Figs 4A and S4A and S4B). Western blotting against the type 1 fimbrial major subunit FimA confirmed a sharp reduction of FimA production by the Δ*ryfA* mutant compared to WT strain CFT073 and the *ryfA* complemented strain (Fig 4B). Surprisingly, although production of type 1 fimbriae was reduced in the *ryfA* mutant, we did not observe significant differences in the RNA-seq data for *fim* genes under growth conditions tested. This could be explained by the fact that at O.D. 0.6, only 4% of the cell population expresses type 1 fimbriae, as previously reported [35]. However, by comparing expression of *fimA* in LB and human urine at mid-log phase and after static growth, by RT-PCR, we observed a significant decrease in *fimA* RNA levels in the *ryfA* mutant (Fig 4C). These results indicate that deletion of the *ryfA* sRNA caused a substantial decrease in the levels of type 1 fimbriae production and *fimA* gene expression.

### Loss of RyfA increases swimming motility

The effect of *ryfA* deletion on motility was determined, since motility contributes to colonization and persistence in the urinary tract [36]. We compared the motility of the *ryfA* mutant in 0.25% soft and urine agar. We reasoned that since the deletion of *ryfA* decreased expression of type 1 fimbriae (Fig 4A), motility of the *ryfA* mutants may potentially be increased. There was a clear and significant increase in motility of the *ΔryfA* strain compared to wild-type CFT073 parent strain in soft agar and urine agar plates (Fig 4D). Interestingly, the upregulation of flagellar and motility genes also corresponds to the increased swimming motility that was observed on motility agar (Fig 3).

### Deletion of *ryfA* decreased the expression of P fimbriae and increased expression of F1C fimbriae

**Fig 5:**
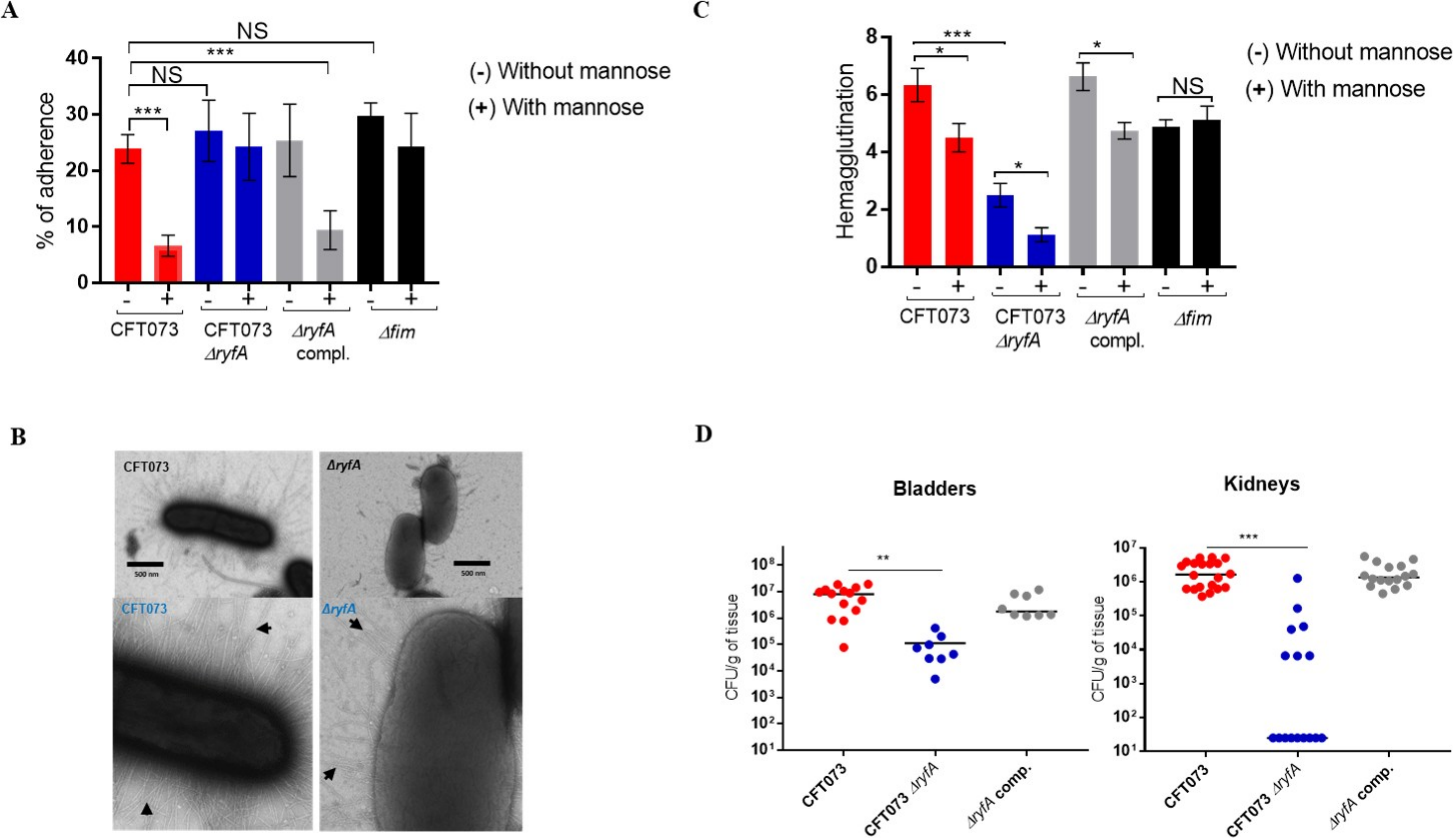
Role of *ryfA* in the murine model of ascending UTI and in P fimbriae (pili) production. (**A**) Adherence of strain CFT073 and its derivatives to human 5637 bladder epithelial cells in the presence or absence of 2.5% α-d-mannopyranose was determined. (**B**) Electron microscopy of CFT073 and the Δ*ryfA* mutant. Arrows show fimbriae on cell surfaces. (**C**) Production of Pap fimbriae by hemagglutination assay. Results are the mean values and standard deviations for four biological experiments. (**D**) Single-strain infections to compare wild-type strain CFT073 to Δ*ryfA* mutant. Results are presented as the log_10_ CFU g^−1^. Each data point represents a sample from an individual mouse, and horizontal bars indicate the medians. Two independent series of infections were performed: **i** (CFT073 WT and CFT073 Δ*ryfA*) and **ii** (CFT073 WT and the complemented mutant). The Δ*ryfA* mutant was attenuated 146-fold in bladder and 10,000-fold in kidneys compared to the WT parent strain. Data are means ± standard errors of the means of 10 mice. Statistical significance was calculated by one-way ANOVA (A and C) or Mann–Whitney Test (D): *, *P* < 0.05; **, *P* < 0.005; ***, *P* < 0.0001. NS, not significant.

It has been observed that deletion of the *fim* operon results in increased expression of other types of fimbriae [13,35]. Since UPEC strains adhere to bladder epithelial cells and epithelial cell adherence is mediated predominantly by type 1 fimbriae, we therefore hypothesized that the *ryfA* mutant may demonstrate reduced adherence to bladder epithelial cells. To test this possibility, adhesion assays using 5637 human bladder epithelial cells (ATCC HTB-9) were performed. The *ryfA* mutant adhered to human bladder cells as well as the WT strain (Fig 5A). As expected, addition of 1.5% α-d-mannopyranose, which inhibits type 1 fimbriae mediated binding, greatly decreased adherence of the WT (mean decrease of 17.2%) and complemented strain (mean decrease of 15.9%) (Fig 5A). By contrast, addition of mannopyranose only slightly reduced adherence of the Δ*ryfA* (mean decrease of 2.8%) and Δ*fim* (mean decrease of 5.4%) mutants, to 5637 bladder cells (Fig 5A). In line with these findings, *fimA* expression was also down-regulated by 4.8-fold in the *ryfA* mutant, during adherence to 5637 bladder cells (S4C Fig).

These results suggest that other adhesins such as the *foc*-encoded F1C fimbriae, which were upregulated in the *ryfA* mutant (Fig 3B), could potentially mediate adherence to the bladder epithelial cells. Further, electron microscopy images demonstrate that the type of fimbriae at the surface of cells of the *ryfA* mutant differ from those produced by the CFT073 wild-type strain and that fimbriae are also somewhat reduced in numbers compared to the WT parent strain (Fig 5B). As such, adherence of the *ryfA* mutant to bladder cells is largely independent of type 1 fimbriae and is mediated by other adhesins present at the cell surface. We also investigated production of Pap fimbriae by mannose-resistant hemagglutination (MRHA) of human erythrocytes of strains cultured in LB broth. Interestingly, the MRHA titer of the *ryfA* mutant was significantly reduced compared to parent strain CFT073 (Fig 5C). This supports the hypothesis that P fimbriae production is also reduced in the *ryfA* mutant. In line with these results, expression of both *papA1* and *papA2* was reduced by 3.5-fold and 6.3-fold, respectively (S4C Fig). These results demonstrate that the mannose-resistant adherence of the *ryfA* mutant to bladder epithelial cells is not attributed to increased production of Pap fimbriae, but could be due to increased production of other adhesins such as F1C fimbriae. It was also previously shown that expression of F1C fimbriae increased considerably in a strain lacking the *fim* and *pap* gene clusters [37]. Thereby, we investigated by qRT-PCR and Western blotting whether expression of F1C fimbriae was increased in this background. As shown in S4C *and* S4D Fig, F1C fimbriae were upregulated 7.9-fold in the *ryfA* mutant. Therefore, upregulation of F1C fimbriae in the *ryfA* mutant could potentially contribute to the *in vitro* adherence of the *ryfA* mutant to bladder cells.

We analyzed the adherence and intracellular bacterial survival of the cystitis isolate UTI89 by using imaging flow cytometry-based assays. The GFP-tagged UTI89 and its derivative strains were allowed to adhere to monolayers of the bladder epithelial cell line 5637 for 2 h (T0), after which extracellular bacteria were killed by addition of the host cell-impermeable antibiotic gentamicin. Following a 4 h (T4) incubation in the continued presence of gentamicin, infected monolayers were washed and intracellular bacterial survival (T4) was quantitatively determined by the percentage of GFP^high^ 5637 cells. Representative images that correspond to single GFP^+^LAMP1^+^ 5637 cells at T0 and T4 are shown in S5 Fig. Interestingly, the percentages of total cell-associated bacteria for Δ*ryfA* and Δ*fim* mutants were significantly reduced (5.03% and 4.5%, respectively) when compared to the WT parent strain (24.36%) (p<0.0001). At 4 h post-infection, results showed a significantly higher percentage of UTI89 GFP^high^ labeled WT bacteria within intracellular compartments compared to either the Δ*ryfA* or Δ*fim* mutants (S5 Fig).

Previous studies showed that the Δ*fim or* Δ*fimH* UTI89 mutant strains were deficient in binding to 5637 cultured bladder epithelial cells *in vitro* and were unable to colonize and invade bladder epithelium by 6 h p.i. relative to the isogenic WT UTI89 control strain [38,39]. The UTI89 Δ*ryfA* mutant also produced less type 1 fimbriae compared to the WT parent strain *in vitro* (S4B Fig). These data suggest that deletion of *ryfA* affects type 1 production in UTI89 and that the adherence to 5637 cells in the absence of the type 1 fimbriae is not sufficient to trigger bacterial internalization into bladder epithelial cells.

### Loss of RyfA reduces virulence and expression of type 1 fimbriae in the murine urinary tract

Based upon increased sensitivity of the Δ*ryfA* mutant to environmental stresses, we hypothesized that the impaired osmotic and oxidative stress response could result in a fitness defect during infection of the urinary tract. In the mouse infection model, the Δ*ryfA* strain showed a marked decrease in bacterial numbers in both bladders (146-fold decrease) (P = 0.0021) and the kidneys (10,000-fold decrease) (P<0.0001) compared to the wild-type strain CFT073 (Fig 5D). The *ryfA* complemented mutant colonized the mouse urinary tract as well as wild-type strain CFT073 (Fig 5D). Likewise, *fimA* transcription was downregulated 5.8-fold in the bladder (Fig 4C). This reduction in colonization from inactivation of *ryfA* could be due in part to the decrease in expression of *fimA* and reduced production of type 1 fimbriae, which was also observed *in vitro* (Fig 4A and 4C). However, it is likely that additional factors including decreased resistance to cellular stresses due to loss of *ryfA* contribute to the strong attenuation and decreased bacterial numbers observed during infection with the Δ*ryfA* mutant, particularly in the kidneys.

In addition, we performed a co-infection of Δ*ryfA* mutant with the virulent CFT073 Δ*lac* strain in the CBA/J murine model. The CFT073 Δ*lac* strain has been shown to colonize the urinary tract as well as the CFT073 wild-type parent and presented no statistical difference in a murine UTI model [40]. Interestingly, the Δ*ryfA* mutant was significantly outcompeted by the CFT073 Δ*lac* strain with a mean of 124-fold in the bladders (p<0.0001) and 13-fold (p<0.0001) in the kidneys (S6A Fig). In contrast to the fitness decrease identified for the Δ*ryfA* strain during UTI, there was no growth difference between CFT073 and CFT073 *ΔryfA* when grown *in vitro* in either LB broth or in filtered human urine. The latter represents an *ex vivo* condition that may reflect nutrient availability and environmental conditions encountered in the bladder (S6B and S6C Fig). The growth of the *ΔryfA* mutant was altered in LB supplemented with 0.6 M urea or 0.6 M NaCl, but not in human urine. Interestingly, concentration ranges of urea (150 mM to 350 mM) and sodium (40 mM to a maximum of 275 mM) in human urine are considerably lower than the concentrations that were inhibitory to the *ryfA* mutant [41–43]. Thus, the competitive defect of the *ryfA* mutant during UTI was not due to a general fitness defect, and that during standard *in vitro* culture conditions, *ryfA* was dispensable. Overall, these results confirm an important role for the RyfA sRNA during colonization and infection of the urinary tract by strain CFT073.

### RyfA contributes to increased uptake and survival of UPEC in professional phagocytes

**Fig 6:**
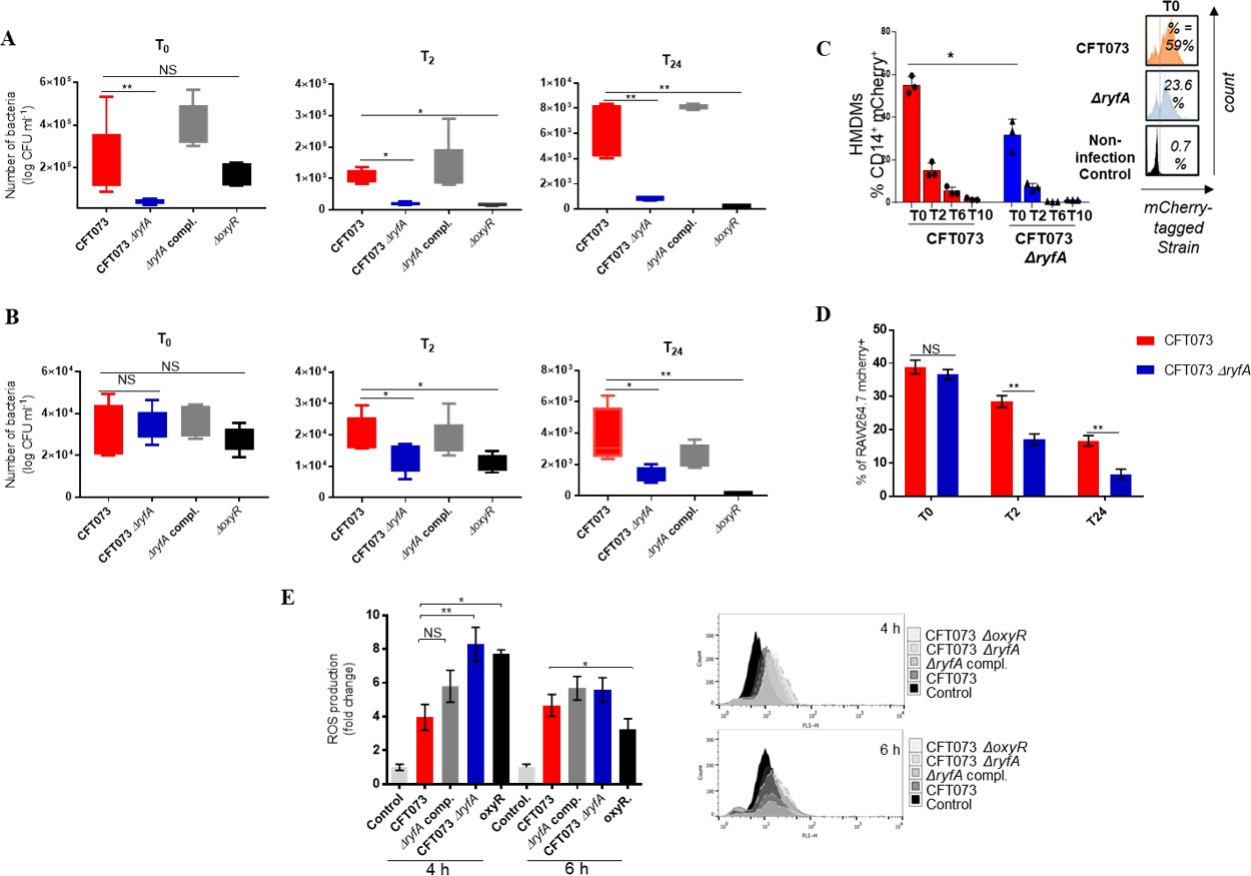
Role of RyfA during interaction with professional phagocytes. Phagocytic cells were infected with CFT073 wild-type strain, Δ*ryfA* or Δ*oxyR* isogenic mutants or the Δ*ryfA*-complemented strain. (**A**) *in vitro* differentiated human monocyte-derived macrophages (HMDMs) and (**B**) THP-1 human macrophages were infected with different strains for 1 h, followed by gentamicin treatment. Cells were lysed and intracellular bacterial counts (CFU ml^−1^) were determined at different times post-infection (pi). Infection assessment of mCherry tagged CFT073 strains via flow cytometry in (**C**) HMDMs and (**D**) RAW264.7 cells. (**E**) Reactive oxygen species (ROS) production in RAW264.7 cells. RAW264.7 cells were infected at an MOI of 20 and intracellular generation of ROS was measured at 4 h and 6 h post-infection by using H_2_DCFDA. Cells were stained with 10 μM H_2_DCFDA. All assays were conducted in triplicate and repeated independently at least three times. The results are expressed as the means ± sem. Significant differences between mutant, WT and complemented mutant strains were determined using One-way ANOVA. *, *P* < 0.05; **, *P* < 0.005; ***, *P* < 0.0001. NS, not significant.

**Fig 7:**
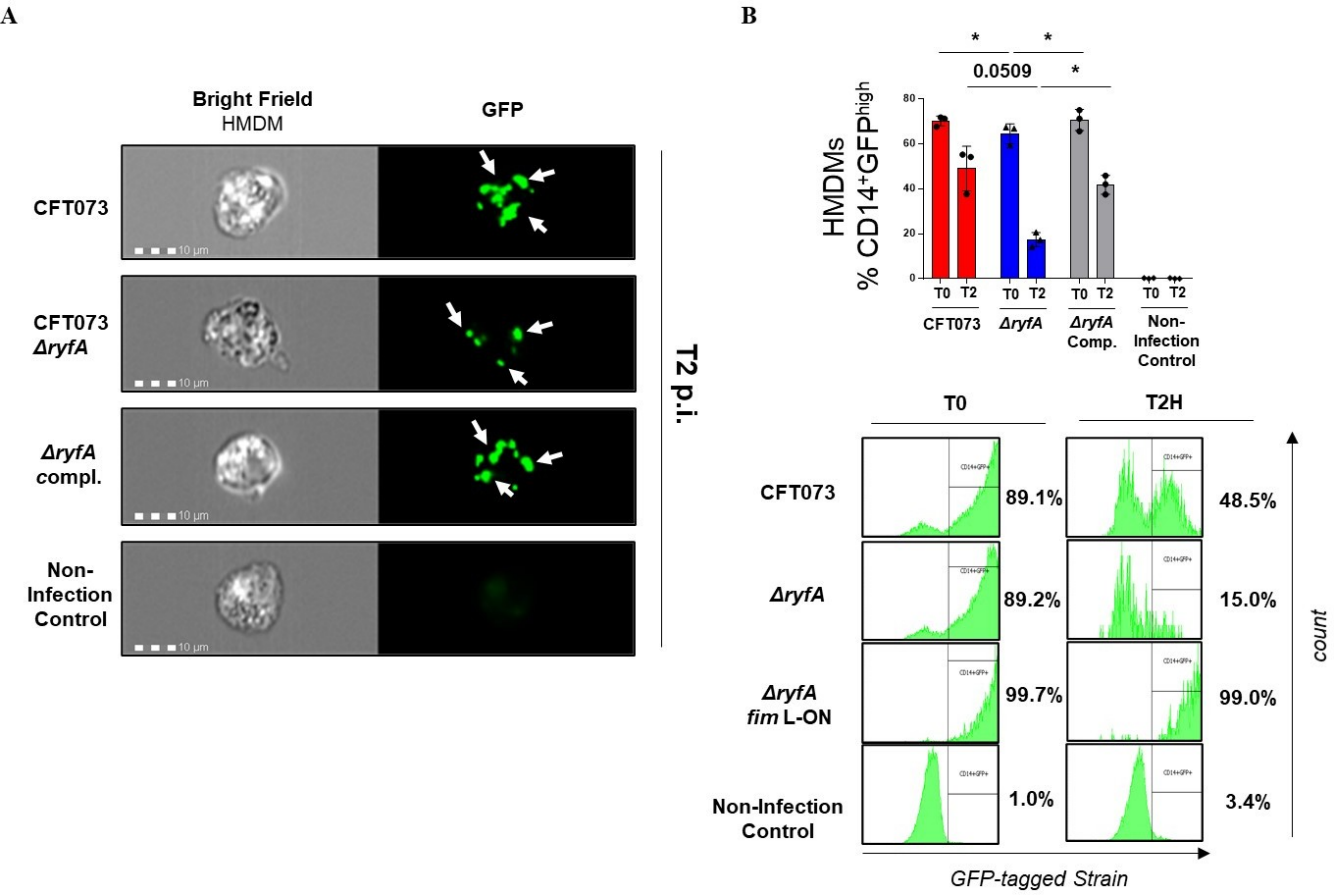
Role of RyfA during interaction with human macrophage cells. Imaging flow cytometry analysis of human monocyte-derived macrophages (HMDMs) that were infected with CFT073 wild-type strain, *ΔryfA* mutant, the *ΔryfA* complemented strain and the *ΔryfA fim* L-ON strain at 2 h post-infection. (**A**) Representative images of HMDM singlets CD14^+^GFP^high^ at 2h post-infection. (**B**) Infection assessment for double positive percentages of CD14^+^GFP^high^ HMDMs. Below, representative flow cytometric histograms for an independent experiment. Results are expressed as the means ± sem of the replicate experiments. Significant differences between mutants, WT, and complemented mutant strain were determined using a paired Student *t*-test. *, *P* < 0.05.

During infection, inflammatory cells produce a variety of antimicrobial factors. A primary antimicrobial agent in this repertoire is superoxide, which reacts with other molecules to form ROS. The primary source of bactericidal ROS during infection is provided by phagocyte oxidase. We next sought to assess the potential role of *ryfA* for protection from the host-generated oxidative environment during UTI by testing bacterial interaction with human macrophages. To evaluate bacterial uptake, survival and proliferation within macrophages, primary human monocyte-derived macrophages (HMDMs) and human cultured macrophage-like THP-1 cells were infected with the wild-type strain and the isogenic mutants using a gentamicin protection assay (at a multiplicity of infection (MOI) of 20). The number of bacteria present at different times was determined by viable counts. We also assessed intramacrophage survival of an *oxyR* mutant for comparison as a sensitive control. The uptake of the *ryfA* mutant was significantly reduced in HMDMs when compared with uptake of the wild-type strain (Figs 6A and 7). However, for the THP-1 macrophages, similar bacterial loads for the different strains were observed (Fig 6B). In contrast to wild-type strain CFT073, for both the *ryfA* and *oxyR* mutants, viable bacteria decreased at 2 h p.i. in all types of macrophages (Figs 6A, 6B and 7). Furthermore, both mutants decreased in the intracellular compartment of macrophages at 24 h p.i. and were unable to reach the number of intracellular bacterial cells observed for the wild-type parent strain. We also obtained similar results by using multi-parameter and imaging flow cytometry (Figs 6D and 7). We monitored the uptake of mCherry-tagged CFT073 wild-type and *ryfA* mutant strains in CD14^+^ HMDMs, which represent a classic phenotype for HMDMs. Results showed a major drop in survival of the *ryfA* mutant in HMDMs compared to the wild-type strain at all-time points (2h, 6 h and 10 h p.i.) (Fig 6C). Furthermore, we confirmed this assessment of the same GFP-tagged CFT073 strains including the *ryfA*-complemented strain via imaging flow cytometry. Representative images were also generated at 2 h p.i. for GFP^high^ labeled strains within intracellular compartments (Fig 7). Interestingly, the Δ*ryfA fim* locked-ON mutant showed a very high internalization level in HMDM compared to the WT CFT073 strain (Fig 7B). This strain also did not present any survival defect at 2 h p.i. (Fig 7B). Previous studies have suggested that fimbriae-mediated adhesion to macrophages helps *E*. *coli* to avoid clearance by the innate immune system [44,45]. In addition, *E*. *coli* overexpressing type 1 fimbriae showed increased intracellular survival in macrophages [46]. Of note, we did not observe a significant difference in the uptake of mCherry-labelled CFT073 and *ryfA* mutant by RAW264.7 macrophages (Fig 6D). However, the *ryfA* mutant survived significantly less than the parent wild-type strain at 2 h and 24 h p.i. in these cells (Fig 6D). These observations demonstrate that loss of *ryfA* can contribute to decreased virulence and clearance during extra-intestinal infections such as UTI, since cells lacking *ryfA* are more susceptible to host phagocytic cells such as macrophages. Importantly, the complemented mutant regained uptake and survival capacity in macrophages similar to wild-type parental strain CFT073 (Figs 6A, 6B and 7). It is of further interest that loss of *ryfA* also resulted in a reduced level of bacterial uptake by phagocytes, suggesting that some of the changes in expression that occur on the bacterial cell surface led to a reduced initial level of phagocytosis by macrophages. This defect of uptake by macrophages by the *ryfA* mutant also is not likely to be linked to increased sensitivity to oxidative stress, since the ROS-sensitive *oxyR* mutant did not demonstrate any differences in uptake by macrophages (Fig 6A and 6B). These results support an important role for the *ryfA* regulatory RNA in interactions with the host cellular response by contributing to uptake, survival, and replication of UPEC CFT073 inside human macrophages.

### The *ryfA* mutant generates increased ROS production in RAW264.7 murine macrophage-like cells

The phagocyte NADPH oxidase and ROS production play a key role in the elimination of bacteria following phagocytosis. To measure intracellular ROS levels, *RAW264*.*7* macrophages infected with *CFT073*, the Δ*ryfA*, Δ*oxyR* isogenic mutant, or the Δ*ryfA* complemented strain were incubated for 4 h and 6 h with the membrane-permeant fluorescent probe H_2_DCFDA. The cells were then stained with a viability marker (propidium iodide) and H_2_DCFDA fluorescence was quantified in viable cells. Four hours later, the level of intracellular ROS after interaction with WT strain CFT073 was 3.9-fold higher when compared to uninfected control cells (p = 0.0026) (Fig 6E). The level of intracellular ROS was 25% (p = 0.0093) and 20% (p = 0.0006) higher in cells infected with the Δ*ryfA* and Δ*oxyR* mutants respectively than in cells infected with the WT parent strain. ROS levels remained high (4-fold higher above basal level) up to 6 h p.i. in cells infected with UPEC CFT073 (Fig 6E). The ROS levels were reduced by 3-fold for the *ryfA* mutant and by half for the *oxyR* mutant after 6 h p.i. (Fig 6E), suggesting that both mutants are killed at a faster rate by ROS activity than the wild-type parent. By contrast, no significant difference was observed between the *ryfA* complemented mutant and the WT parent strain in ROS generation. These results also support the likelihood that attenuation of the *ryfA* mutant in the bladder and kidneys during UTI in the mouse model may also be due to a greater sensitivity to killing by host phagocytic cells and an increased generation of ROS within these cells.

### Effects due to loss of *ryfA* in UPEC are independent of a predicted small peptide sequence

The mechanisms of interaction of *ryfA* and its specific role in the adaptive response of pathogenic *E*. *coli* to resist environmental stresses and infect the urinary tract remain to be elucidated. A recent study identified that the *ryfA* gene from *Salmonella enterica* serovar Typhimurium can encode a small toxic inner membrane protein, called TimP, upon overexpression [27]. Alignment of the *ryfA* sequence of UPEC CFT073 also identified an open reading frame (ORF) spanning nt +135 to +270 (S7A Fig). This ORF is predicted to be translated into a 45-amino-acid small protein (S7B Fig). We also identified a putative Sec-dependent secretion signal using two different prediction tools: SignalP-5.0 [47] and Phobius [48] and both programs predicted a Sec translocase signal sequence spanning amino acids 1 to 22 (S7B Fig). In order to determine if such a peptide could potentially contribute to some of the regulatory changes observed due to deletion of *ryfA*, we introduced different point mutations that would eliminate the potential production of such a peptide. Three different variants of *ryfA* wherein the *E*. *coli* ORF predicted to encode a paralog of TimP was disrupted were generated, since some nucleotide changes could potentially affect the secondary structure of the RNA (S8 Fig). We then introduced these variants in single copy into the chromosome to complement the *ryfA* mutant of strain CFT073. Complementation of the Δ*ryfA* mutant with any of the 3 variants of RyfA restored production of type 1 fimbriae (S7C Fig), reduced motility (S7D Fig) to wild-type levels, led to a regain in resistance to oxidative stress (S7E Fig) and survival in THP-1 macrophages (S7F Fig). These results suggest that the effects observed due to deletion of *ryfA* in UPEC do not involve the loss of a peptide that it may encode, but are more likely to be linked to the RyfA sRNA.

## Discussion

Given the importance of sRNAs for bacterial physiology and virulence, we investigated the role of the RyfA sRNA for resistance to stresses and its importance during urinary tract infection in a murine model, as well as for adherence to bladder epithelial cells, and survival in human macrophages. RyfA was shown to contribute to resistance to oxidative and osmotic stresses (Figs 1 and S1), virulence in the urinary tract of mice, and survival in host cells. Attenuation of the *ryfA* mutant could be attributed in part to increased sensitivity to osmotic and oxidative stresses, both of which are likely to be encountered during UTI. Further, downregulation of type 1 fimbriae in the *ryfA* mutant may also contribute to reduced colonization during UTI. Deletion of *ryfA* decreased production of type 1 fimbriae or *fim* gene expression *in vitro* and *in vivo* (Figs 4A and 4C and S4A). It is interesting that there is a link between sensitivity to oxidative stress and altered expression of type 1 fimbriae. Previous studies reported that mutations in other genes, including *yeaR*, *rpoS*, *ibeA* and *yqhG*, increased sensitivity to oxidative stress and concomitantly decreased expression of type 1 fimbriae [28,49–51]. Importantly, assembly of type 1 fimbriae is dependent on the oxidative state of subunit proteins in the periplasm and involves the coordinated folding of subunits through action of the oxidoreductase DsbA and the chaperone FimC, underscoring the potential effects of oxidative imbalances on fimbrial biogenesis [52]. Collectively, these results suggest that one consequence of loss of *ryfA* may be the reduced expression of type 1 fimbriae due to increased sensitivity of UPEC to oxidative stress.

Based on the RNA-seq data obtained during growth *in vitro*, the *ryfA* mutant may be subjected to increased oxidative stress, because it is less able to deploy mechanisms to mediate a global stress response, which may also explain why it is less able to cope with additional stresses from exogenous ROI-generating compounds. The general stress response regulon under control of *rpoH*, the oxidative stress transcriptional factor *soxS*, and genes responding to heat shock and envelope stress were all repressed in the *ryfA* mutant (S1 Table). SoxS and MarA are transcription factors that contribute to adaptive responses to many stimuli, such as pH, antibiotics, oxidative stress, and host innate immune responses [53,54]. Furthermore, a triple knockout of a UPEC strain lacking *marA*, *soxS* and *rob* was unable to effectively colonize the kidney in a mouse model [55]. Since oxidative stress is increased during infection of the host, a decreased capacity to resist ROS could explain, at least in part, the attenuation of the *ryfA* mutant.

In the urinary tract, UPEC must be able to cope with transient exposure to both high osmolality and the denaturing effects of urea. RyfA contributed to resistance to osmotic stress induced by NaCl or urea (Fig 1A). Indeed, genes involved in the osmotic stress response, the glycine betaine and proline transporter *proVW*, were downregulated in the *ryfA* mutant (Fig 3). The *proVW* genes are induced in response to osmotic stress with NaCl [56] and during urinary tract infection [37] in the mouse model. Loss of *ryfA* may increase sensitivity to osmotic stress due to reduced expression of the *proVW* system. Other genes such as molecular chaperones encoding *ibpA*, *ibpB*, *cspF*, and *cspH*, which are induced in response to osmotic stress [56], were also repressed in the *ryfA* mutant. Interestingly, it was previously reported that *cspC* and *ibpB* genes were highly expressed in UPEC CFT073 isolated from mouse urine [57]. Further, *cspA*, *cspG*, *cspH*, *ibpA* and *ibpB* were among the most highly upregulated genes in asymptomatic bacteriuria (ABU) strains during static growth in human urine [58]. Moreover, the two cold-shock-associated genes *cspA* and *deaD*, were upregulated in human urine samples [59], by UPEC in murine bladders *[37]* and in a ABU strain in the human urinary tract [37,60]. Taken together, the decreased capacity of the *ryfA* mutant to express these stress coping chaperones and osmotic stress systems is likely to contribute to its reduced survival during UTI. Genes belonging to the Cys regulon, *soxS* and *ibpA* were reported to contribute to potassium tellurite (K_2_TeO_3_) resistance [61]. Interestingly, the *ryfA* mutants of UPEC strains were more sensitive to K_2_TeO_3_ (S1C Fig). The oxidative damage attributed to potassium tellurite is due to the intracellular generation of superoxide radicals.

UPEC strain CFT073 contains 12 distinct fimbrial gene clusters and multiple nonfimbrial adhesins [62]. However, one type of fimbria is predominantly expressed at a time, and phase variation of different adhesins involves crosstalk between fimbrial systems that limits production of fimbriae to a single type for each bacterial cell [63]. Based on gene expression data and immune-based detection, we determined that F1C fimbrial production was increased in the *ryfA* mutant (S4C and S4D Fig). Since F1C fimbriae mediate adhesion to bladder and kidney epithelial cells [64], this likely explains the mannose-resistant adherence demonstrated by the *ryfA* mutant (Fig 5A). Increased expression of F1C fimbrial genes was also observed in a *Δfim Δpap* mutant of UPEC CFT073 [13]. Indeed, in UPEC, the inactivation or constitutive expression of one fimbrial system can alter the expression of other adhesins [13]. Thus, it is not surprising that loss of *ryfA* in UPEC CFT073, which caused a decreased expression of type 1 and P fimbriae, has resulted in greater expression of another type of fimbrial adhesin. Taken together, these results demonstrate that *ryfA* also plays an important role in regulation and the hierarchy of cross-talk of expression of different fimbrial systems in *E*. *coli*.

The CFT073 Δ*ryfA* mutant adhered to human bladder cells as well as the WT strain. However, in contrast to the WT strain, which adhered mainly due to mannose-specific type 1 fimbriae, adherence of the *ryfA* mutant was mediated by mannose-resistant adhesins (Fig 5A). Electron microscopy demonstrated a difference in the type of fimbriae present at the cell surface of the *ryfA* mutant compared to the WT strain (Fig 5B), indicating that other adhesins are expressed at the bacterial cell surface and could mediate adherence to bladder epithelial cells. Further, the *ryfA* mutant had reduced MRHA compared to the WT strain (Fig 5C), and the expression of both *papA* genes was decreased during adhesion to bladder cells (S4C Fig). Interestingly, *ryfA* inactivation in the highly invasive cystitis strain UTI89 also significantly decreased the adherence and invasion capacity of the mutant to 5637 bladder epithelial cells (S5 Fig). Type 1 fimbriae production was also decreased in the UTI89 *ryfA* mutant (S4B Fig).

Bacterial virulence factors are often secreted or associated with the cell envelope. In the *ryfA* mutant, many genes encoding cell envelope associated proteins demonstrated significant changes in expression. We observed significant up-regulation of genes encoding flagellar biosynthesis (*flgCBGD* and *fliC*) and chemotaxis (*cheW*) (Fig 3 and S1 Table) and other genes under the control of σ^28^, encoded by FliA (RpoF) which controls transcription of a number of genes involved in flagellar assembly and swimming motility. Down-regulation of *fim*, *pap*, *f9* and *yad* fimbrial transcription could also be linked to an increase in flagellar gene expression, since the *ryfA* mutant demonstrated increased swimming motility (Fig 4D), and also decreased expression of P and type 1 fimbriae (Figs 4A and 5C). The production of P fimbriae is coordinated with the repression of swimming motility [65]. Furthermore, Luterbach and Mobley [65] have demonstrated that PapX, a MarR-like protein encoded by the *pap* operon, repressed *flhD* transcription and the Δ*papX* mutant was hypermotile. Taken together, these observations indicate that type 1, P, and F1C fimbriae, along with motility, can be regulated in some coordinated fashion through a mixture of transcriptional and posttranscriptional mechanisms. Of note, motility in the urinary tract can be important for the translocation of bacteria from bladder to kidneys. In contrast to our results, a previous report on an extraintestinal pathogenic *E*. *coli* (ExPEC) strain isolated from an ocular infection (L-1216/2010) demonstrated reduced swimming motility compared to the parent strain [24].

In the current work, the *ryfA* mutants of CFT073 and 536 exhibited a significant decrease in biofilm formation in M9 supplemented with glucose at different temperatures and in LB (at 37 and 42°C for CFT073 *ΔryfA* and at 30 and 37°C for 536 *ΔryfA*) (S9 Fig). Accordingly, we also noted a statistically significant downregulation of some of the genes involved in biofilm formation, including, genes important for cellulose synthesis (*bcsS)*, curli fiber production (*csgB)* and lipid A biosynthesis (*lpxP)*. Further, *ibpA* and *ibpB* genes encoding small heat shock chaperone proteins which were downregulated in our mutant, were found to be induced during biofilm formation [66], and *ibpB* was also highly expressed by UPEC CFT073 isolated from mouse urine [57]. Therefore, these heat shock proteins may also promote resistance to stress by contributing to biofilm formation.

Macrophages employ ROS and RNS, that can contribute to bacterial killing [67]. In this study, the *ryfA* mutant showed significantly reduced uptake by human monocyte derived-macrophages (HMDMs) (Figs 6A and 7) as well as decreased survival rates in HMDMs, THP-1 and RAW264.7 cell lines when compared to the WT strain at all time points (Figs 6 and 7). These results indicate that RyfA plays an important role for survival in macrophages. The decreased survival inside macrophages could be explained by the numerous defects of the *ryfA* mutant, such as increased sensitivity to oxidative stress. Interestingly, the *ryfA* mutant induced high levels of intracellular ROS in RAW264.7 macrophages at 4 h p.i compared to WT strain CFT073 (Fig 6E), which may explain why the *ryfA* mutant survives less inside macrophages. Thus, a common mechanism used by bacterial pathogens to avoid host innate immune pathways is to employ defense mechanisms against oxidative stress [68]. Interestingly, genes associated with intramacrophage survival including chaperones (*ibpA*, *ibpB*), *trxC*, *phoH* and *soxS* were downregulated in the *ryfA* mutant. Further, genes that were highly expressed and which were shown to be important for persistence of UPEC strain UTI89 within murine macrophages [69] including sigma factor H (*rpoH*), the phage shock proteins (*pspACDE*), DNA damage inducible protein (*yebG*), and small heat shock protein (*ibpB*), and biofilm formation regulatory protein (*bssS*) were all repressed in the *ryfA* mutant. The Psp system senses membrane stress and stabilizes the bacterial cell membrane under stress conditions [70]. This system is induced in *Shigella flexneri* infecting macrophage [71]. Interestingly, we found that *pspA*, *rpoH*, *ibpA* and *soxS* where all significantly downregulated in the *ryfA* mutant after 6 h p. i. in HMDM (S10 Fig). A previous study showed that *S*. *dysenteriae* harbors two copies of RyfA that contribute to multiplication of *S*. *dysenteriae* in host cells. Specifically, the over-expression of RyfA1 negatively impacted the virulence-associated process of cell-to-cell spread by elimination of *ompC* mRNA that encodes the major outer membrane protein C [26]. We report here that deletion of *ryfA* did not affect the growth of the mutant in LB broth or in human urine (S6B and S6C Fig). This lends support to our assertion that the observed defect in colonization by the *ryfA* mutant is likely due to an increased sensitivity to certain environmental stresses and changes in metabolic functions that could lead to alterations in production of fimbriae/flagella and reduced survival during infection.

Interestingly, *ryfA* RNA was significantly higher in mouse bladder after 48 h p.i. (4.82-fold) and after static growth in LB (2.44-fold) and human urine (2.03-fold) (S3B Fig). By analyzing the transcriptome profile of a UTI strain isolated directly from patients, *ryfA* was also shown to be upregulated in *E*. *coli* asymptomatic bacteriuria ABU strain 83972 by 1.5 to 8.9-fold compared to bacteria grown in MOPS or urine *in vitro* [60]. The ABU strain is an excellent colonizer of the human urinary tract, where it causes long-term bladder colonization.

Although, many sRNAs have been shown to mediate regulation at the RNA level through pairing with other RNA transcripts, it deserves mention that some bacterial sRNAs may code for small peptides. There are several examples of sRNAs which code for validated functional small proteins: *E*. *coli* SgrS encodes the protein SgrT [72], *S*. *aureus* RNAIII encodes a 26 amino acid δ-hemolysin peptide [73] and *B*. *subtilis* SR1 encodes 39 amino acid peptide SR1P [74]. A recent study also identified that the *ryfA* gene from *Salmonella enterica* serovar Typhimurium can encode a small toxic inner membrane protein, called TimP, upon overexpression [27]. The authors also suggested a potential ORF paralog in other Enterobacteria including *E*. *coli* [27]. To investigate if such a peptide contributed to the phenotypes observed in the *ryfA* mutant, we derived variants of the *ryfA* allele wherein this ORF predicted to encode such a peptide was eliminated. Introduction of any of these variant alleles in the Δ*ryfA* mutant fully complemented the mutant phenotypes (S7 Fig). As such, these experiments indicated that the potential peptide that could be encoded by *ryfA* in *E*. *coli* did not play an appreciable role in the regulatory effects observed due to loss of the *ryfA* gene. In the experiments presented in [27], these researchers reported effects on *Salmonella* due to artificially induced or high-level expression, and it is unknown under what natural conditions in the bacterial cell, this TimP peptide might be expressed. Notably, Fris et al. [26] investigated the characterization of *ryfA* in *Shigella dysenteriae* and attempted to identify putative peptides that might be encoded by *ryfA* and did not demonstrate production of any potential small protein under conditions tested.

Taken together, several lines of evidence suggest that inactivation of *ryfA* could attenuate pathogenic *E*. *coli* by altering regulatory pathways leading to changes in metabolism and decreased adaptation to environmental stresses or may potentially generate an altered response to membrane-associated stress, leading to repression of production of surface structures, including adhesins required for colonization of host tissues. Based on the important number of adaptive and virulence associated genes that are under the control of the RyfA sRNA, it should be considered as a potential target for therapeutic interventions or preventative measures against UPEC and potentially other enterobacterial pathogens.

## Methods

### Ethics statement

This study was performed in accordance with the ethical standards of the University of Quebec, INRS. A protocol for obtaining biological samples from human donors was reviewed and approved by the ethics committee—*Comité d’éthique en recherche* of INRS (CER 19–507, approved November 19, 2019). Formal written consent was obtained by the donors.

### Bacterial strains, growth conditions and plasmids

*E*. *coli* strains and plasmids used in this study are listed in S2 Table. *E*. *coli* CFT073 was initially isolated from the blood and urine of a patient with acute pyelonephritis [75]. Bacteria were grown in lysogeny broth (LB) (Alpha Bioscience, Baltimore, MD) at 37°C and in human urine. Urine was collected from healthy female volunteers that were from 20 to 40 years old and who had no history of UTI or antibiotic use in the prior 2 months. Each urine sample was immediately filter sterilized (0.2-μm pore size), pooled, and frozen at −80°C and was used within 2 weeks. Antibiotics and reagents were added as required at the following concentrations: kanamycin, 50 μg/ml; ampicillin, 100 μg/ml; chloramphenicol 30 μg/ml.

### Construction of site-directed mutants and complementation of strains

All mutants were generated by the procedure described by Datsenko and Wanner using plasmids pKD3 and pKD4 as the template for chloramphenicol and kanamycin resistance cassettes, respectively [76]. Primers used are listed in S3 Table in the supplemental material. Antibiotic resistance cassettes flanked by FLP recombination target (FRT) sequences were removed by transforming the mutant strains with pCP20 expressing the FLP recombinase [77].

### Growth under conditions of osmotic stress

Strains were tested for the capacity to grow under conditions of osmotic stress caused by NaCl or urea. Strains were inoculated 1:100 from an overnight pre-culture grown in LB and grown until mid-log phase with shaking. They were serially diluted and plated on LB agar alone and LB agar supplemented with 0.6 M NaCl or 0.6 M urea. Colonies were counted, and growth under each condition was compared to growth on LB agar.

### Sensitivity of *E*. *coli* strains to reactive oxygen intermediate (ROI)-generating agents

Sensitivity to oxidative stress generating agents was determined by an agar overlay diffusion method on LB plates (1.5% agar) as described by Sabri *et al*. [78]. Briefly, overnight-grown cultures were used to inoculate (1/100) fresh LB medium without antibiotics, and the resulting cultures were incubated until the O.D. _600_ was 0.6. Then, 100 μl of each culture were mixed with 3 ml molten top agar and poured onto an LB agar or M9- glucose plate. Whatman filter disks saturated with 10 μl of hydrogen peroxide (30%), plumbagin (53 mM) or paraquat (40 mM) were spotted onto the disks. The plates were then incubated overnight at 37°C. Following growth, the diameters of inhibition zones were measured.

For sensitivity of bacterial cultures to H_2_O_2_, bacteria were grown at 37°C in LB broth and approximately 2.5 x 10^8^ CFU/ml of wild-type CFT073 or mutant was inoculated into PBS containing either 5 mM H_2_O_2_ and or H_2_O. The test was carried out under static conditions at 37°C and samples were collected at various time points post-inoculation. Samples were diluted and plated on LB agar to determine bacterial counts.

### Experimental UTI in CBA/J mice

Experimental infections were carried out using either competitive coinfection or single-strain infection models as described previously [40]. Prior to inoculation, strains were grown for 16 h at 37°C with shaking (250 rpm) in 55 ml of LB medium. For coinfection, cultures were centrifuged and pellets of a *lac*-negative derivative of the wild-type (WT) and mutant or complemented strains were mixed 1:1. Six-week-old CBA/J female mice were transurethrally inoculated with 20 μl of the 1:1 mixture containing 2 × 10^9^ CFU of the UPEC CFT073 Δ*lacZYA* strain (QT1081) and 5 × 10^8^ CFU of either the CFT073 Δ*ryfA* (QT5255) strain or its complemented derivative (QT5309). At 48 h p.i., mice were euthanized; bladders and kidneys were aseptically removed, homogenized, diluted, and plated on MacConkey agar to determine bacterial counts. In the single-strain experimental UTI model, mice were infected as described above but with only a single strain (10^9^ CFU), and 48 h p.i., bacterial counts were determined from the bladders and kidneys. Bladders were dissected; one half was used to determine bacterial counts and the other half was resuspended in TRIzol reagent (Invitrogen) for RNA extraction and subsequent analysis of bacterial gene expression.

### RNA extraction and quantification of gene expression

Bacterial cultures were grown in triplicate in LB broth or human urine. RNAprotect (Qiagen, Toronto, ON, Canada) was added to cultures and RNA extracted using the RNeasy mini kit (Qiagen, Toronto, ON, Canada). Ambion Turbo DNase (Thermo Fisher Scientific, St. Laurent, QC, Canada) was used to remove contaminating DNA. RNA integrity was assessed using a Nanodrop (ND-1000). RNA was also extracted from infected bladders at 48 h p.i. with TRIzol reagent (Thermo Fisher Scientific, St. Laurent, QC, Canada), followed by DNase 1 treatment. Total RNAs were then reverse-transcribed to cDNAs using TransScript All-in-One First-Strand cDNA Synthesis SuperMix Kit (TransGen, Haidian District, Beijing, China). The resulting cDNA was used for qRT PCR with EvaGreen according to the manufacturer’s instructions (TransGen, Haidian District, Beijing, China). The *rpoD* gene was used as housekeeping control. Each qRT-PCR run was done in triplicate, and for each reaction the calculated threshold cycle (*C*_*T*_) was normalized to the *C*_*T*_ of the *rpoD* gene amplified from the corresponding sample. The fold change was calculated using the 2^−ΔΔ^*CT* method [79]. Genes with a fold-change above or below the defined threshold of 2 were considered as differentially expressed. Primers used for qRT-PCR analysis are listed in S3 Table in the supplemental material.

### RNA sequencing, mapping and analyses

Cultures were grown in triplicate as described above, and total RNA was isolated using the Qiagen RNeasy Protect Bacteria Mini kit. RNA-seq was performed at Génome Québec Innovation Centre, McGill University. Total RNA was quantified using a NanoDrop Spectrophotometer ND-1000 (NanoDrop Technologies, Inc.) and its integrity was assessed on a 2100 Bioanalyzer (Agilent Technologies, St. Laurent, QC, Canada). rRNA were depleted from 250 ng of total RNA using Ribo-Zero rRNA removal kit specific for bacterial RNA (Illumina). Residual RNA was cleaned up using the Agencourt RNA Clean XP Kit (Beckman Coulter) and eluted in water. cDNA synthesis was achieved with the NEB Next RNA First Strand Synthesis and NEBNext Ultra Directional RNA Second Strand Synthesis Modules (New England BioLabs, Whitby, ON, Canada). The remaining steps of library preparation were done using and the NEBNext Ultra II DNA Library Prep Kit for Illumina (New England BioLabs, Whitby, ON, Canada). Adapters and PCR primers were purchased from New England BioLabs, Whitby, ON, Canada. Libraries were quantified using the Quant-iT PicoGreen dsDNA Assay Kit (Thermo Fisher Scientific, St. Laurent, QC, Canada) and the Kapa Illumina GA with Revised Primers-SYBR Fast Universal kit (Kapa Biosystems, Wilmington, MA, USA). Average size fragment was determined using a LabChip GX (PerkinElmer, Woodbridge, ON, Canada) instrument (Génome Québec).

Reads were checked for quality using the FASTQC tool (Galaxy Version 0.72+galaxy1), poor quality reads by Trimmomatic (Galaxy Version 0.38.0) were converted to FastQ format, or “groomed” for downstream analysis using the FastQ Groomer tool (Galaxy Version 1.1.5). Bowtie (Galaxy Version 2.3.4.3+galaxy0) was used to align RNA-seq reads to the genome of *E*. *coli* CFT073 (GenBank accession no. AE014075.1 with default parameters. Finally, the number of reads mapping to each annotated feature was obtained with HTSeq (Galaxy Version 0.9.1). Differential expression between WT CFT073 and the *ryfA* mutant was analyzed using Degust v4.1.1 (DOI: 10.5281/zenodo.3258932). Transcripts were considered significant if passing the following cut-offs: adjusted p-value of < 0.05 and log_2_ fold change of >1.7 and <-1.7. All processing steps were performed using the Galaxy platform (https://usegalaxy.org/).

RNA-seq data have been deposited in the GEO database, accession number GSE157450 (https://www.ncbi.nlm.nih.gov/geo/query/acc.cgi?acc=GSE157450).

### Verification of RNA-seq results (validation of RNA-seq results by qPCR)

RNA samples were obtained and prepared as described above for RNA-seq. qRT-PCR was performed to verify the RNA levels of 8 genes selected for validation (*soxS*, *ibpA*, *cspA*, *marA*, *bssS*, *rpoH*, *cadA* and *treC*).

### Northern blot analysis

The hot phenol method was used to extract total RNA [80]. A sample of 10 μg of total RNA was loaded on a 5% acrylamide (29:1)/8M urea gel. RNA was electro-transferred to a Hybond-XL membrane (Amersham Biosciences) and UV-crosslinked (1200J). Prehybridization was done in 50% formamide, 5× SSC, 5× Denhardt reagent, 1% SDS, and 100 μg/ml sheared salmon sperm DNA for 4 h at 60°C. The radiolabeled RNA probe was added and incubated overnight. Three 15 minutes washes of the membranes were made with 1× SSC/0.1% SDS followed by a final wash with 0.1× SSC/0.1% SDS at 65°C.

### RNA probe radio-labeling

The RNA probe antisense to the RyfA sRNA was synthesized *in vitro* using the T7 RNA polymerase. Oligos EM4969B and EM4970 were used to generate the transcription template. Transcription was carried out in the T7 transcription buffer (40 mM Tris-HCL at pH 8.0, 6 mM MgCl2, 10 mM dithiothreitol, 2 mM spermidine), 400 μM NTPs (A, C and G), 10 μM UTP, 3 μl of α-32P-UTP (3000 Ci/mmol), 20 U RNA guard, 20 U T7 RNA polymerase and 0.5 μg DNA template. After 2 h of incubation at 37°C, the mixture was treated with 2 U of Turbo DNAse (Ambion) for 15 minutes. The radio-labeled probe was then purified on a G50-Sephadex column before hybridization.

### Evaluation of type 1 and P fimbriae production

The level of production of type 1 fimbriae was determined by a yeast agglutination assay [35]. Briefly, the strains were cultured at 37°C / 250 rpm in LB broth or human urine to mid-log phase (conditions which were used for transcriptional analyses), or static in LB broth, M9-glucose or human urine overnight. Following centrifugation, 40 μl of an initial suspension of approximately 2 × 10^11^ cells ml^−1^ in PBS was transferred and serially diluted 2-fold in microtiter wells containing equal volumes of a 3% commercial yeast suspension in PBS. After 30 min of incubation on ice, yeast aggregation was monitored visually. The agglutination titer was defined as the most diluted sample showing agglutination.

The mannose-resistant hemagglutination (MRHA) assay was determined using human type O^+^ and A^+^ erythrocytes to determine HA that could be mediated by P fimbriae. Bacterial strains were grown to mid-log phase or passaged six times on LB agar at 37°C, after which a bacterial sample was transferred to a well containing 50 μl of 3% (vol/vol) human red blood cells. To inhibit type 1 fimbriae-dependent hemagglutination, a final concentration of 2.5% α-d-mannopyranose was added to samples.

### Preparation of fimbrial extracts and Western blotting

Preparation of fimbrial extracts and Western blotting were performed as described previously [81], with anti-FimA serum from *E*. *coli* strain B_AM_ and F1C fimbriae-specific (anti-F165_2_) antiserum [82].

### Adherence and gentamicin protection (invasion) assays

5637 human bladder cells (ATCC HTB-9) were grown in RPMI 1640 medium (Wisent Bioproducts) supplemented with 10% fetal bovine serum, 2 mM l-glutamine, 10 mM HEPES, 1 mM sodium pyruvate, 4.5 g/liter glucose, and 1.5 g/liter sodium bicarbonate. 5637 cells were grown to confluency in RPMI 1640 and 2 × 10^5^ cells/well were distributed in 24-well plates. UPEC CFT073 and its derivative strains were grown in LB medium at 37°C to the mid-log phase of growth (O.D. 0.6). Immediately before infection, cultures were washed once with PBS to remove dead cells, returned to the infection medium, and infected at an estimated MOI of 10 CFU per cell. The bacterial cells were centrifuged, washed twice with PBS, resuspended in RPMI 1640 medium (Wisent Biocenter, St-Bruno, Canada) supplemented with 10% fetal bovine serum at 10^6^ CFU ml^−1^, and added to each well. Bacterium-host cell contact was enhanced by a 5-min centrifugation at 600 × *g*. After 2 h, cells were washed three times and lysed with PBS–0.1% sodium deoxycholate (DOC), serially diluted, and plated on LB agar plates. Quantification of cell-associated bacteria was performed. To block adherence mediated by type 1 fimbriae, 2.5% α-d-mannopyranose was added to culture medium.

For GFP-tagged UTI89, bacteria were grown at 37°C in LB broth supplemented with ampicillin to O.D. 0.6. Cells were infected with UTI89 using a MOI of 10 bacteria per host cell. After a 2 h incubation at 37°C, samples were washed three times with PBS to remove any non-adherent bacteria. Triplicate samples were used to calculate the total number of the associated bacteria present both intra- and extracellularly. To determine invasion frequencies, after the initial 2 h incubation, additional sets of wells were washed three times with PBS and then incubated for another 2 h in medium containing 100 μg/ml membrane-impermeable bactericidal antibiotic gentamicin to kill any extracellular bacteria. Following additional washes with PBS, fresh medium containing a lower concentration of gentamicin (10 μg/ml) was added, and incubations were continued for another 2 h. This submaximal concentration of gentamicin was used to prevent extracellular growth of UPEC while limiting possible leaching of the antibiotic into the host cells during longer incubations. Monolayers were then washed with PBS and the positivity percentage of GFP-tagged bacteria present within the cells were measured by using ImageStream-based assay.

### Electron microscopy

Cells for electron microscopy were grown as described above for agglutination assay experiments. A glow-discharged Formvar-coated copper grid was placed under a drop of bacterial culture for 15 min to allow cells to adsorb. Excess liquid was removed using filter paper, just before a drop of 1% phosphotungstic acid (negative stain) was placed onto the grid for 15 sec. Samples were left to air dry and viewed using a Hitachi H-7100 transmission electron microscope.

### Swimming motility assay

Motility assays were done using UPEC strains CFT073 and 536 and its mutant derivatives as previously described [28,36] with modifications. Following overnight growth at 37°C, strains were cultured at 37°C in LB broth to mid-log phase. Soft agar (1% tryptone, 0.5% NaCl, 0.25% agar) or urine agar, were stabbed in the center of the plate using an inoculating needle. Care was taken not to touch the bottom of the plate during inoculation to ensure only swimming motility was assessed. After 16 h (soft agar) or 22 h (urine agar) of incubation at 37°C, the motility diameters were measured for each strain. Wild-type *E*. *coli* CFT073 was always included as a reference, and five independent motility experiments for each mutant were performed. *E*. *coli* CFT073 *fim L-ON* was used as a negative control. Results were analyzed using a paired t-test.

### Infection of human cultured macrophage-like cell line THP-1

The human monocyte cell line THP-1 (ATCC TIB-202) was maintained in RPMI 1640 (Wisent, Saint-Jean-Baptiste, QC, Canada) containing 10 % (v/v) heat-inactivated FBS (Wisent, Saint-Jean-Baptiste, QC, Canada), 1 mM sodium pyruvate (Wisent, Saint-Jean-Baptiste, QC, Canada) and 1 % modified Eagle’s medium with non-essential amino acids (Wisent). A stock culture was maintained as monocyte-like, non-adherent cells at 37°C in an atmosphere containing 5 % (v/v) CO_2_. The bacterial strains were cultured at 37°C in LB broth to mid-log phase. Bacterial cells were pelleted, washed with phosphate buffer saline and added to the cell monolayer at a MOI of 20, and plates were centrifuged for 5 min at 800 ×*g* to synchronize bacterial uptake. After 60 min of incubation at 37°C, extracellular bacteria were removed by washing cells three times with prewarmed PBS, and the infected monolayers were either lysed with PBS-DOC (T0) or incubated for 2 h or 24 h with medium containing 100 μg gentamicin ml^–1^ (Wisent) to kill remaining extracellular bacteria, and then with 12 μg gentamicin ml^–1^ for the remainder of the experiment. Surviving bacteria were determined (CFU) by plating on LB agar. Results are expressed as the mean±sem of at least three experiments performed in duplicate. One-way ANOVA was used for statistical analysis.

### Human monocyte-derived macrophage cultures

Peripheral blood mononuclear cells (PBMC) were obtained from leukaphereses of healthy donors. All participants of the study received approval from the McGill University Health Centre Ethical Review Board (ethic reference number SL-00.069). At least a purity of 96% of human monocytes was obtained using the Human Monocyte Enrichment Kit (Stem Cell Technologies, Vancouver, BC). Afterwards, purified human monocytes were seeded in RPMI 1640 medium supplemented with 2mM glutamine, antibiotics, and 10% FBS in the presence of 50 ng/ml of Human recombinant Macrophage colony-stimulating factor (M-CSF). After 6 days, human monocyte-derived macrophages (HMDMs) were used for infection assays. Differentiation follow-up during 6 days was assessed by flow cytometry analysis.

### Multiparametric and imaging flow cytometry analyses

To assess bacterial infection in macrophages, HMDMs were harvested after adding non-enzymatic cell dissociation solution (Sigma-Aldrich, Oakville, ON, Canada) for 30 mins at room temperature. Cells were washed with PBS and prepared in FACS tubes. For surface staining, we used the following specific monoclonal antibodies provided from BD Biosciences for phenotypic analysis of human HMDMs: anti-CD3 (BV605), anti-CD14 (BV650), anti-CD80 (PE Cy7), anti-CD209 (PE) and anti-CD64 (PE). Finally, the viability marker 7-aminoactinomycin D or 7-AAD (ThermoFisher Scientific, St. Laurent, QC, Canada) was used to exclude dead cells from analyses. Afterwards, cells were fixed with 4% paraformaldehyde solution and then washed. For data analysis, samples were analyzed by flow cytometry with a BD LSRII Fortessa flow cytometer and DIVA software (BD). Viable gated cell singlets were analyzed for each sample and the percentages of mCherry^high^ CFT073 containing CD14^+^ HMDMs were determined. For Imaging flow cytometry, the same procedures were applied while cells were prepared in Eppendorf tubes. For surface staining, we used CD14-V450 and CD80-APC H7 for phenotypic gating of HMDMs. Samples were acquired using the Image Stream X MKII flow cytometer and analyzed with IDEAS software (Amnis) and representative images of singlets CD14^+^ HMDMs were generated expressing GFP^high^ CFT073 bacterial cells.

### Measurement of ROS production

RAW264.7 cells were infected at an MOI 20:1 in complete DMEM medium in 24-well plates. The same conditions as for infection of THP-1 cells were used. After 60 min of incubation at 37°C, extracellular bacteria were removed by washing cells three times with prewarmed PBS, and the cells were then incubated for 4 h or 6 h with medium containing 100 μg gentamicin ml^–1^ to kill extracellular bacteria. The fluorescent probe 2′, 7′-dichlorodihydrofluorescein diacetate (H_*2*_DCF-DA) (ThermoFisher Scientific, St. Laurent, QC, Canada), was solubilized at 5 mM in dimethyl sulfoxide (DMSO) (Sigma). Infected or uninfected cells were incubated with 10 μM H_2_DCFDA for 45 min at 37°C, and then washed once with PBS 1X. Cells were then trypsin treated, washed once with PBS 1X and cell pellets were suspended in a 1 μg/ml propidium iodide solution (ThermoFisher Scientific, St. Laurent, QC, Canada). The flow cytometer FACSCalibur (Becton Dickinson) was used to measure the fluorescence of the oxidized product dichlorofluorescein at a λ of 488 nm with excitation at 530 nm. The fluorescence of propidium iodide was measured at a λ of 585 nm with an excitation at 542 nm. A total of 10 000 viable cells were analyzed by sample. For all points, experiments were performed in replicates. Statistical differences between different conditions were determined with a two-tailed Student’s *t* test on the indicated number (*n*) of experiments.

### Biofilm assay

Biofilm formation on polystyrene surfaces was assessed in 96-well plates. Strains were grown at various temperatures (25°C, 30°C, 37°C, and 42°C) for 48 h under static conditions in LB and M9 supplemented with 0.2% glucose. Wells were washed and stained with 0.1% crystal violet (Millipore Sigma) for 15 min, then 200 μl of ethanol-acetone (80:20) solution was added, followed by measuring at an optical density at OD_595_.

### Site-directed mutagenesis to generate *ryfA* variants containing point mutations

Site-directed mutagenesis was performed using the Q5EvaGreen Site-Directed Mutagenesis kit as specified by the manufacturer (New England Biolabs). pIJ546 was used as a template for the construction of the RyfA variants with specific mutations pIJ588, pIJ589 and pIJ590 at 25 to 50 ng per reaction with 10 pmol of each of the complementary primers. Primers used to generate the point mutations were CMD 2712 and CMD2713 for pIJ588 (variant 1), CMD 2715 and CMD2716 for pIJ589 (variant 2) and CMD2718 and CMD2719 for pIJ590 (variant 3). Following mutagenesis, all constructs were verified by PCR and by sequencing.

### Statistical analyses

Statistical analyses were performed using the Prism 7.04 software package (GraphPad Software). Statistically significant differences between two groups were established by unpaired t-test and comparisons among three or more groups was done by one-way analysis of variance (ANOVA). For the independent infections, comparisons of the CFU mL−1 or CFU g−1 distributions were analyzed using the Mann–Whitney test. A Wilcoxon signed-rank test (two-tailed; *P* ≤ 0.05) was used to determine statistical significance for comparison of bacterial numbers in coinfection experiments.

## Supporting information

S1 Fig Role of RyfA in stress resistance. (**A**) Comparison of growth characteristics of CFT073, Δ*ryfA* mutant and Δ*ryfA* complemented strain diluted and plated on LB agar after growth in LB at 37°C to a O.D 0.6. (**B**) Growth inhibition zones (mm) of UPEC 536 and the *ryfA* mutant to ROI-generating compounds on LB and M9-glucose agar. (**C**) Sensitivities of UPEC strains CFT073 and 536 and their derivative strains to potassium tellurite on LB agar plates. Tests were performed as described in Methods. The results represent the means of replicate experiments for a minimum of three samples. Vertical bars represent the standard errors of the means. Statistical significance was calculated by one-way ANOVA (B and C): *, *P* < 0.05; **, *P* < 0.005; ***, *P* < 0.0001.
(TIF)

S2 Fig RyfA predicted structure and sequence. (**A**) Schematic depicting the chromosomal location of *ryfA* in *E*. *coli* CFT073 and other species. (**B**) Clustal Alignment of RyfA alleles from *E*. *coli* K-12 and 3 UPEC strains. The 304 nucleotide sequence is based on the *E*. *coli* K-12 MG1655 reference allele. Overall, 21 variable nucleotides were present, and the *E*. *coli* K-12 allele had one gap at nucleotide 290 compared to the UPEC strains. UPEC CFT073 was more similar to MG1655 as these alleles only varied at 6 sites (including the gap). UTI89 and 536 were highly similar to each other with only 3 differences. Alignment generated using MEGAX software (https://www.megasoftware.net). (**C**) The Vienna RNA websuite was used to predict the secondary structure of RyfA from CFT073. The structure is colored by base-pairing probabilities.
(TIF)

S3 Fig *ryfA* expression at different conditions and genes affected by the absence of *ryfA in vitro*. (**A**) Validation of RNA-seq data by qRT-PCR. RNA was isolated from UPEC CFT073 and the *ΔryfA* mutant in mid-log growth (O.D. 0.6) in LB at 37°C and qRT-PCR analysis was performed. Genes either upregulated or downregulated by at least 2-fold were considered significant. (**B**) Expression of *ryfA* gene in the WT CFT073 strain in infected bladders and after static growth in LB broth or human urine compared to RNA levels compared to expression when grown to mid-log growth (O.D. 0.6) in LB at 37°C. qPCR data represent means of relative expression ± range (*n*  =  3) of three biological replicates (* p < 0.05, **p < 0.01, ***p < 0.001 using one-way ANOVA).
(TIF)

S4 Fig Effect of inactivation of *ryfA* on production of type 1 fimbriae (pili) and expression of Pap and F1C fimbriae (pili). Type 1 fimbriae production determined by yeast agglutination. The level of type 1 fimbriae production in UPEC strains. (**A**) CFT073 grown static overnight in M9 medium containing 0.2% glucose, (**B**) 536, UTI89, and derivatives after mid-log growth in LB or overnight in human urine. (**C)** qRT-PCR analysis of *fimA* (Type 1), *papA* (*papA1* and *papA2-*P fimbriae) and *focA* (F1C fimbriae) genes from CFT073 Δ*ryfA* mutant adhering to 5637 bladder cells compared to levels for WT strain. The dashed line corresponds to the cutoff for a significant difference in expression. All results shown are the mean values and standard deviations for four biological experiments. Statistical significance was calculated by the one-way ANOVA (A, B and C): *, *P* < 0.05; **, *P* < 0.005; ***, *P* < 0.0001. (**D**) Western blot of fimbrial extracts using F1C-specific (anti-F165_2_) antiserum. The *fim*-negative *E*. *coli* K-12 strain ORN172 carrying the plasmid pYVAN which expresses F1C (F165_2_) fimbriae was used as positive control. Bands are from a representative gel.
(TIF)

S5 Fig Effect of loss of *ryfA* on adherence and intracellular bacterial survival of UPEC strain UTI89. 5637 bladder epithelial cell monolayers were infected for 2 h with UPEC UTI89 and its derivatives strains (MOI: 10) using ImageStream-based assay. After 2 h p.i (T0), cells were then washed four times with PBS and total cell-associated bacteria were determined. After 2 h p.i., 5637 bladder epithelial cell monolayers infected with UTI89 were incubated for 4 h in medium containing gentamicin to prevent extracellular bacterial growth and to allow time for the establishment of UPEC within the host bladder cells (T4). We included non-infected cells as a negative experimental control. (**A**) Representative images of single GFP^+^LAMP1^+^ 5637 cells at T0 and T4. (**B**) UPEC infection measurement was determined by the percentage of GFP^high^ 5637 cells at different time points p.i.. LAMP1, Lysosomal-associated membrane protein 1. Data represent the mean results ± SEM from three or more independent assays performed in triplicate. Statistical significance was calculated by one-way ANOVA (A, B and C): *, *P* < 0.05; **, *P* < 0.005; ***, *P* < 0.0001.
(TIF)

S6 Fig Inactivation of *ryfA* in uropathogenic *E*. *coli* CFT073 reduces competitive colonization of the mouse urinary tract. (**A**) Co-infection experiments between a CFT073 Δ*lac* and Δ*ryfA* mutant. Data are means ± standard errors of the means of 10 mice (**B**) Growth curves of CFT073 and *ryfA* mutant in LB broth (**C**) Comparison of growth characteristics of CFT073 or CFT073 *Δlac* and *ryfA* mutant in human urine in monoculture and coculture. There were no significant differences in growth between CFT073 and *ryfA* mutant in all conditions tested. Error bars represent the SEM. *P* < 0.05; **, *P* < 0.005; ***, *P* < 0.000 Mann–Whitney Test.
(TIF)

S7 Fig The predicted TimP paralog in the *E*. *coli ryfA* gene does not contribute to phenotypes observed in the *ryfA* mutant. (**A**) DNA sequence of the *ryfA* CFT073 predicted ORF corresponding to TimP is indicated with a green arrow. (**B**) The predicted small protein from RyfA carries a putative Sec system signal sequence in CFT073 (shown in red). (**C**) Type 1 fimbriae production determined by yeast agglutination in strains cultured to the mid-log phase of growth in LB broth and O/N in urine and (**D**) Motility in 0.25% soft agar of CT073, isogenic *ryfA* mutant and the different variant complemented mutants. (**E**) Growth inhibition zones (mm) of CFT073 and its derivative strains to oxidative stress generating compounds (30% H_2_O_2_) on LB agar. (**F**) THP-1 human macrophages were infected (MOI: 20) with different strains for 1 h, followed by gentamicin treatment. Cells were lysed and intracellular bacterial counts (CFU ml^−1^) were determined at 2 h p.i. Data represent the averages of at least three separate experiments. Error bars represent the SEM. Statistical significance was calculated by one-way ANOVA: *, *P* < 0.05; **, *P* < 0.005; ***, *P* < 0.0001. NS, not significant.
(TIF)

S8 Fig *ryfA* alleles with point mutations introduced to eliminate the potential TimP *E*. *coli* paralog peptide. (**A**) The predicted peptide potentially associated with the RyfA RNA from CFT073. Native ORF present in *E*. *coli* CFT073 (**B**) Variant *ryfA* alleles wherein small sequence changes would eliminate or alter the ORF through introduction of stop codons or frame-shifts. These three variants of *ryfA* would not contain the predicted ORF and could not produce such a peptide.
(TIF)

S9 Fig Biofilm formation in UPEC strains CFT073 and 536 and respective *ryfA* mutants. UPEC and *Serratia liquefaciens* strain were grown at different temperatures (30°C, 37°C, and 42°C) in LB and minimal M9 medium containing 0.2% glucose in polystyrene plate wells for 48 h and then stained with crystal violet. Remaining crystal violet after washing with acetone was measured as absorbance at 595 nm. Data are the means of three independent experiments, and error bars represent standard errors of the means. The *Serratia liquefaciens* strain was used as positive control for biofilm formation. *, *P* < 0.05; **, *P* < 0.005; ***, *P* < 0.0001 compared to CFT073 or 536 using one-way ANOVA.
(TIF)

S10 Fig Genes whose expression is reduced in the absence of *ryfA* during infection of human macrophages. UPEC genes associated with intramacrophage survival. HMDMs were infected at an MOI of 20. Intracellular bacterial survival was assessed at 6 h post-infection and the relative quantity of mRNA of specific genes were determined by qRT-PCR. qPCR data represent means of relative expression ± range (*n* =  3) of three biological replicates (* p < 0.05, **p < 0.01, ***p < 0.001 using one-way ANOVA). Results were normalized against the steady state RNA levels of *rpoD* (see Methods for experimental details).
(TIF)

S1 Table Examination of transcriptome of CFT073 vs *ΔryfA* mutant in LB at O.D 0.6. Strains were inoculated in triplicate 1:100 from an overnight pre-culture grown in LB and grown until mid-log phase with shaking (250 rpm).
(PDF)

S2 Table Bacterial strains and plasmids used in this study. (DOCX)

S3 Table Primers used in this study. (PDF)
