# Supplementary figures and images for: The RyfA small RNA regulates oxidative and osmotic stress responses and virulence in uropathogenic Escherichia coli
Source: PLoS Pathog. 2021 May 27;17(5):e1009617. doi: 10.1371/journal.ppat.1009617 (PMC8205139; doi:10.1371/journal.ppat.1009617)

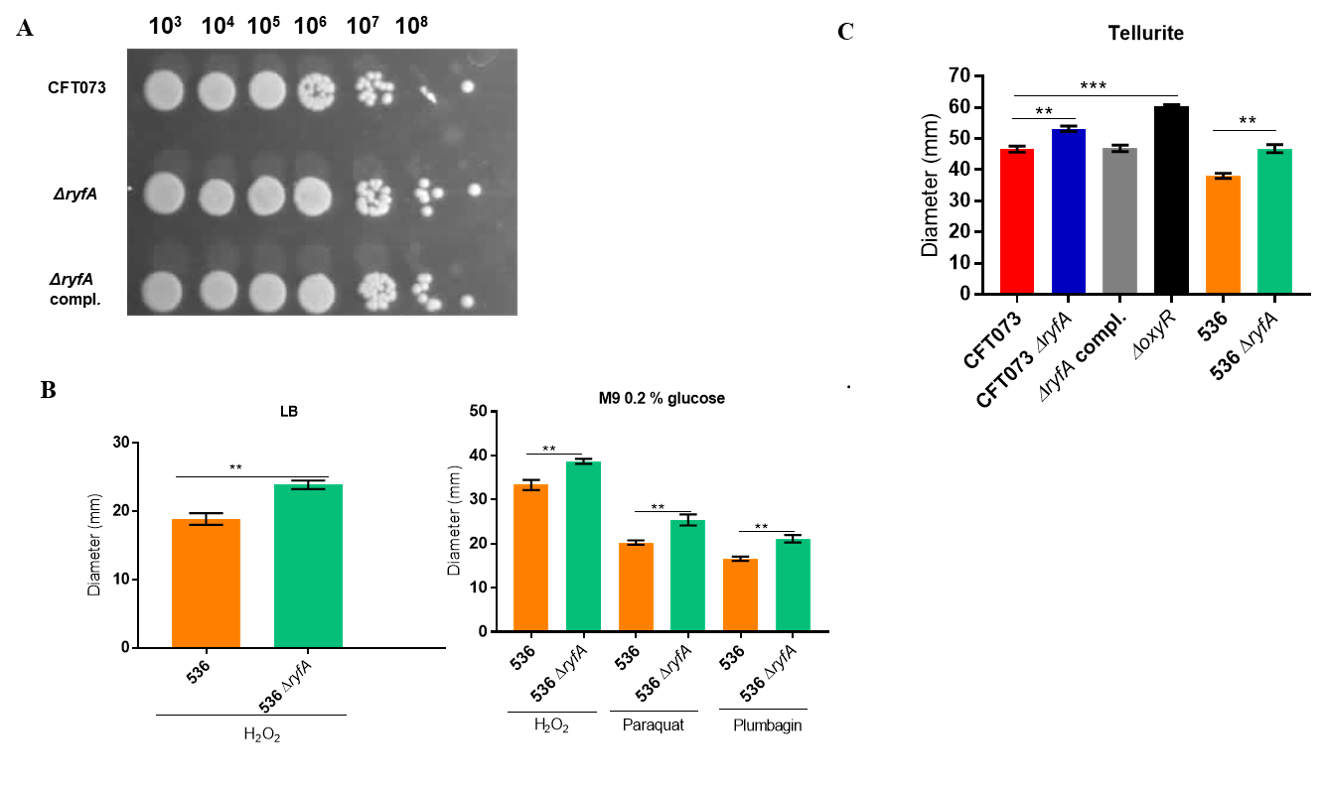

Supplement: S1 Fig — (A) Comparison of growth characteristics of CFT073, ΔryfA mutant and ΔryfA complemented strain diluted and plated on LB agar after growth in LB at 37°C to a O.D 0.6. (B) Growth inhibition zones (mm) of UPEC 536 and the ryfA mutant to ROI-generating compounds on LB and M9-glucose agar. (C) Sensitivities of UPEC strains CFT073 and 536 and their derivative strains to potassium tellurite on LB agar plates. Tests were performed as described in Methods. The results represent the means of replicate experiments for a minimum of three samples. Vertical bars represent the standard errors of the means. Statistical significance was calculated by one-way ANOVA (B and C): *, P < 0.05; **, P < 0.005; ***, P < 0.0001. (TIF) [file ppat.1009617.s001.tif]

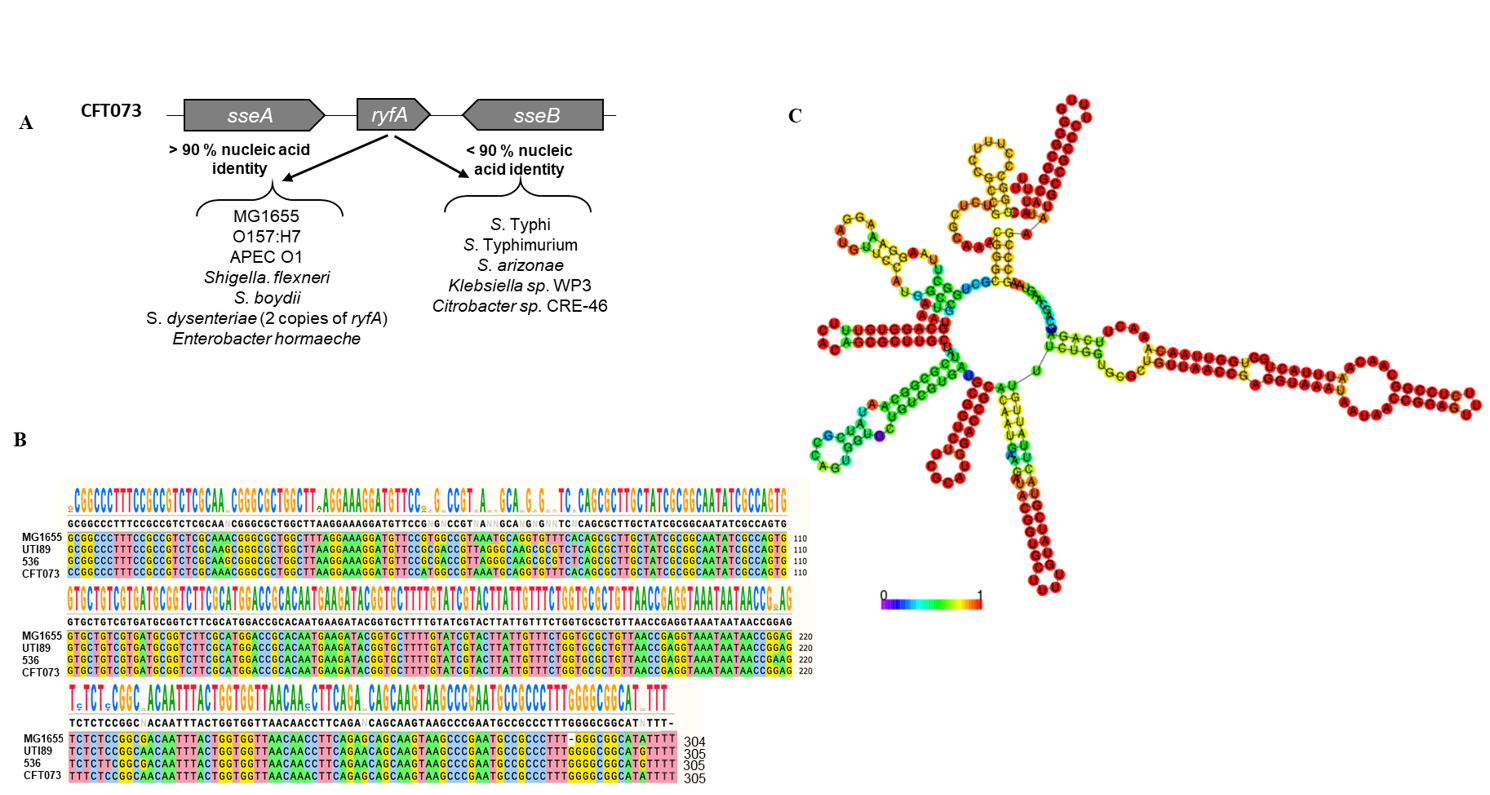

Supplement: S2 Fig — (A) Schematic depicting the chromosomal location of ryfA in E. coli CFT073 and other species. (B) Clustal Alignment of RyfA alleles from E. coli K-12 and 3 UPEC strains. The 304 nucleotide sequence is based on the E. coli K-12 MG1655 reference allele. Overall, 21 variable nucleotides were present, and the E. coli K-12 allele had one gap at nucleotide 290 compared to the UPEC strains. UPEC CFT073 was more similar to MG1655 as these alleles only varied at 6 sites (including the gap). UTI89 and 536 were highly similar to each other with only 3 differences. Alignment generated using MEGAX software (https://www.megasoftware.net). (C) The Vienna RNA websuite was used to predict the secondary structure of RyfA from CFT073. The structure is colored by base-pairing probabilities. (TIF) [file ppat.1009617.s002.tif]

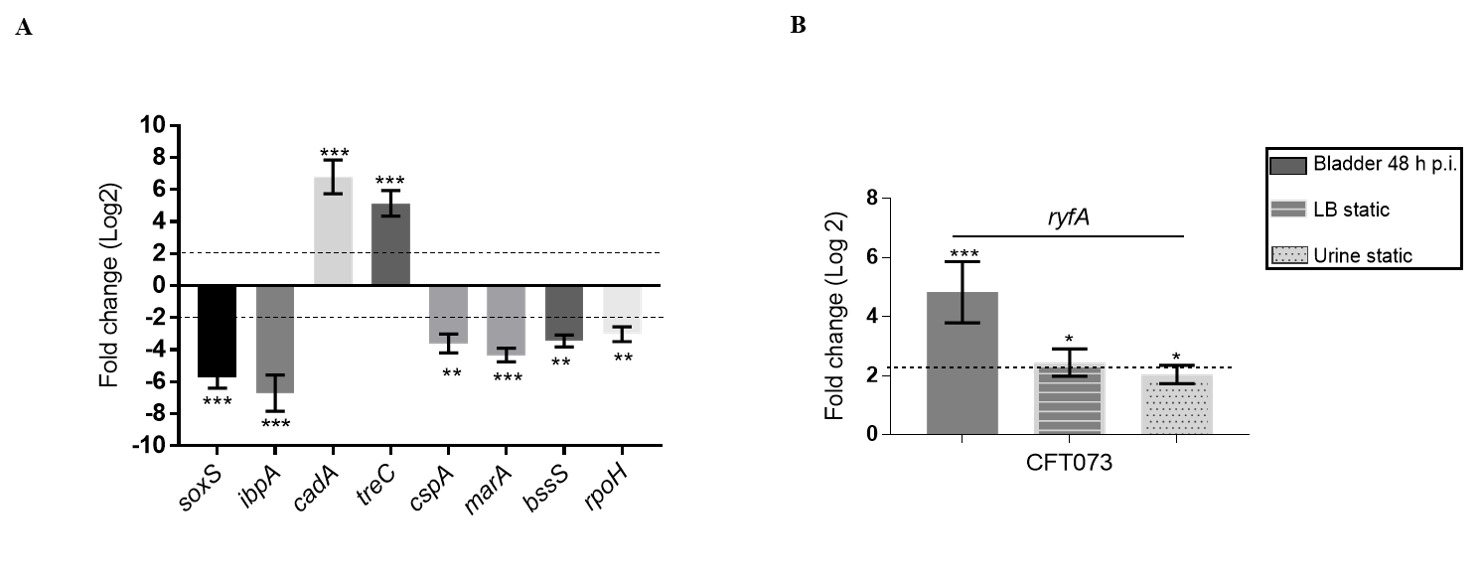

Supplement: S3 Fig — (A) Validation of RNA-seq data by qRT-PCR. RNA was isolated from UPEC CFT073 and the ΔryfA mutant in mid-log growth (O.D. 0.6) in LB at 37°C and qRT-PCR analysis was performed. Genes either upregulated or downregulated by at least 2-fold were considered significant. (B) Expression of ryfA gene in the WT CFT073 strain in infected bladders and after static growth in LB broth or human urine compared to RNA levels compared to expression when grown to mid-log growth (O.D. 0.6) in LB at 37°C. qPCR data represent means of relative expression ± range (n = 3) of three biological replicates (* p < 0.05, **p < 0.01, ***p < 0.001 using one-way ANOVA). (TIF) [file ppat.1009617.s003.tif]

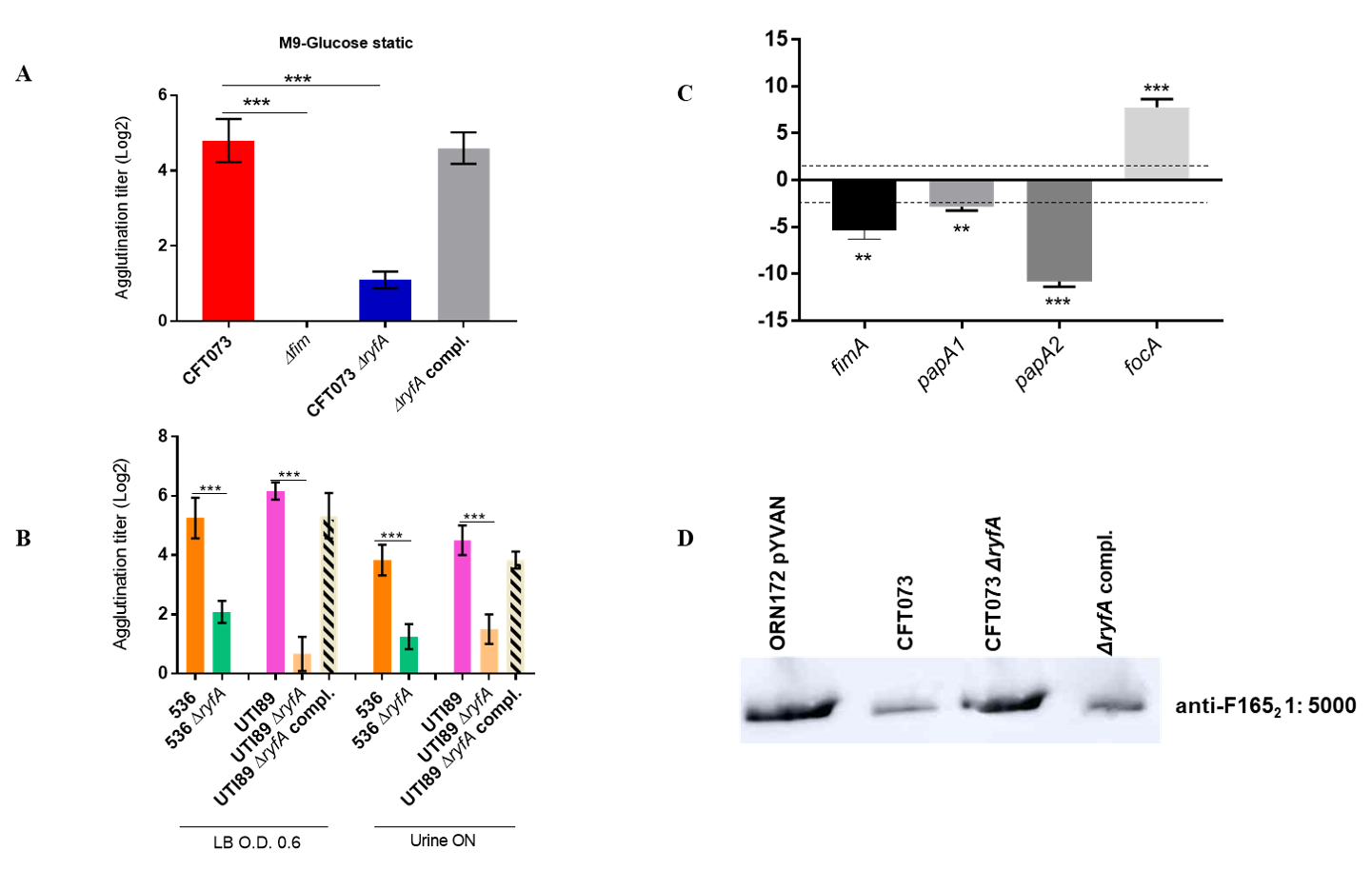

Supplement: S4 Fig — Type 1 fimbriae production determined by yeast agglutination. The level of type 1 fimbriae production in UPEC strains. (A) CFT073 grown static overnight in M9 medium containing 0.2% glucose, (B) 536, UTI89, and derivatives after mid-log growth in LB or overnight in human urine. (C) qRT-PCR analysis of fimA (Type 1), papA (papA1 and papA2-P fimbriae) and focA (F1C fimbriae) genes from CFT073 ΔryfA mutant adhering to 5637 bladder cells compared to levels for WT strain. The dashed line corresponds to the cutoff for a significant difference in expression. All results shown are the mean values and standard deviations for four biological experiments. Statistical significance was calculated by the one-way ANOVA (A, B and C): *, P < 0.05; **, P < 0.005; ***, P < 0.0001. (D) Western blot of fimbrial extracts using F1C-specific (anti-F1652) antiserum. The fim-negative E. coli K-12 strain ORN172 carrying the plasmid pYVAN which expresses F1C (F1652) fimbriae was used as positive control. Bands are from a representative gel. (TIF) [file ppat.1009617.s004.tif]

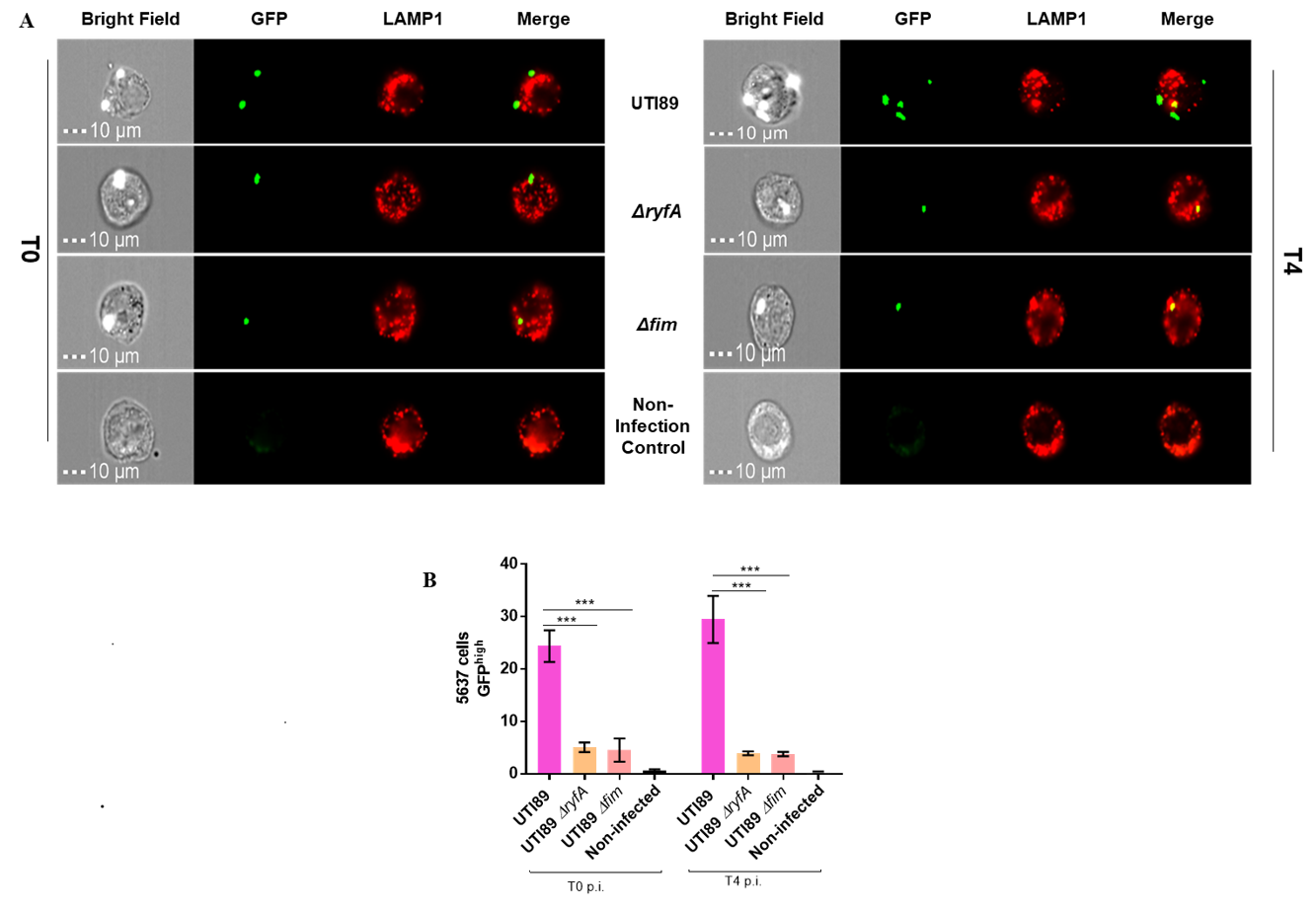

Supplement: S5 Fig — 5637 bladder epithelial cell monolayers were infected for 2 h with UPEC UTI89 and its derivatives strains (MOI: 10) using ImageStream-based assay. After 2 h p.i (T0), cells were then washed four times with PBS and total cell-associated bacteria were determined. After 2 h p.i., 5637 bladder epithelial cell monolayers infected with UTI89 were incubated for 4 h in medium containing gentamicin to prevent extracellular bacterial growth and to allow time for the establishment of UPEC within the host bladder cells (T4). We included non-infected cells as a negative experimental control. (A) Representative images of single GFP+LAMP1+ 5637 cells at T0 and T4. (B) UPEC infection measurement was determined by the percentage of GFPhigh 5637 cells at different time points p.i.. LAMP1, Lysosomal-associated membrane protein 1. Data represent the mean results ± SEM from three or more independent assays performed in triplicate. Statistical significance was calculated by one-way ANOVA (A, B and C): *, P < 0.05; **, P < 0.005; ***, P < 0.0001. (TIF) [file ppat.1009617.s005.tif]

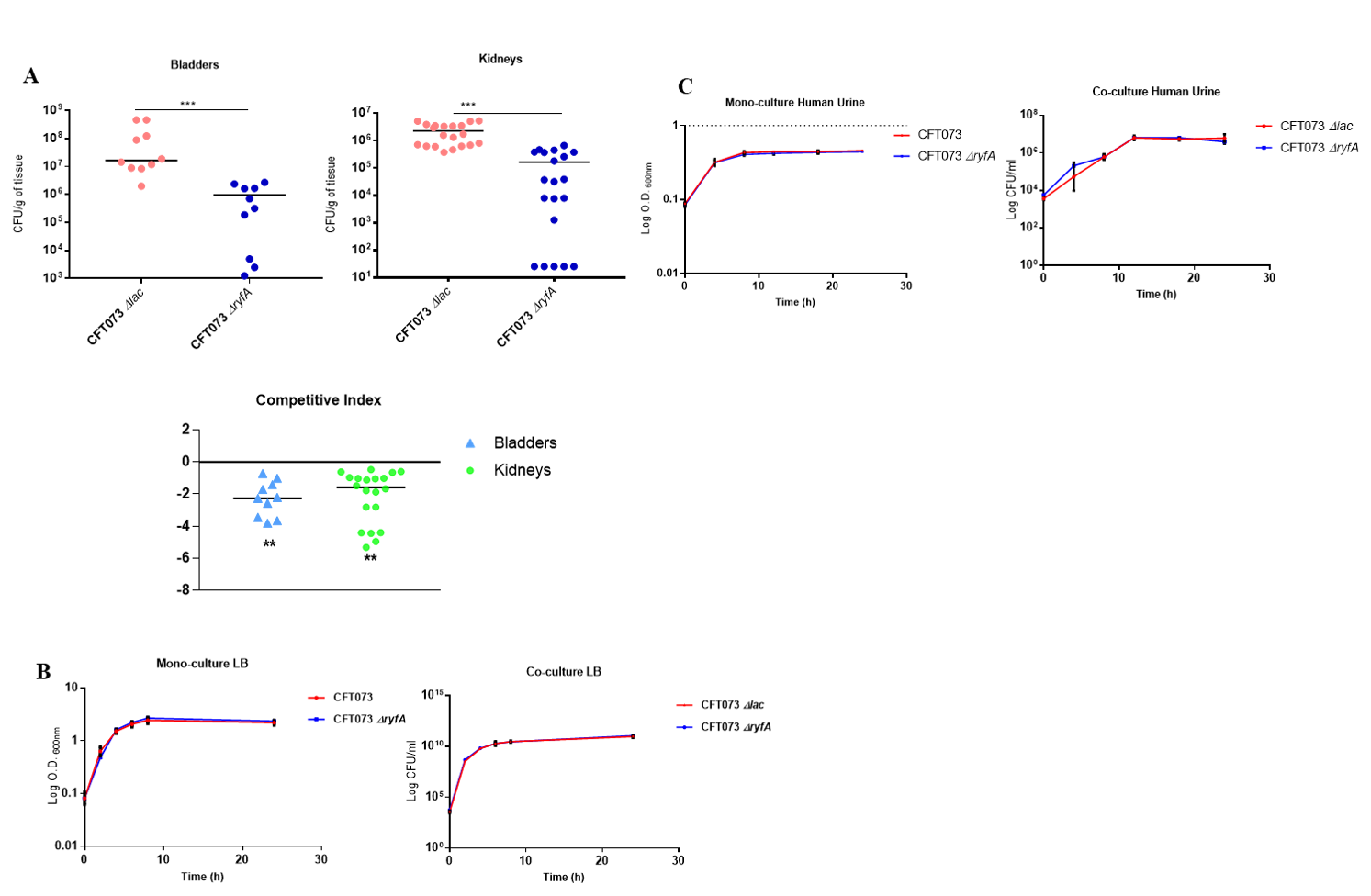

Supplement: S6 Fig — (A) Co-infection experiments between a CFT073 Δlac and ΔryfA mutant. Data are means ± standard errors of the means of 10 mice (B) Growth curves of CFT073 and ryfA mutant in LB broth (C) Comparison of growth characteristics of CFT073 or CFT073 Δlac and ryfA mutant in human urine in monoculture and coculture. There were no significant differences in growth between CFT073 and ryfA mutant in all conditions tested. Error bars represent the SEM. P < 0.05; **, P < 0.005; ***, P < 0.000 Mann–Whitney Test. (TIF) [file ppat.1009617.s006.tif]

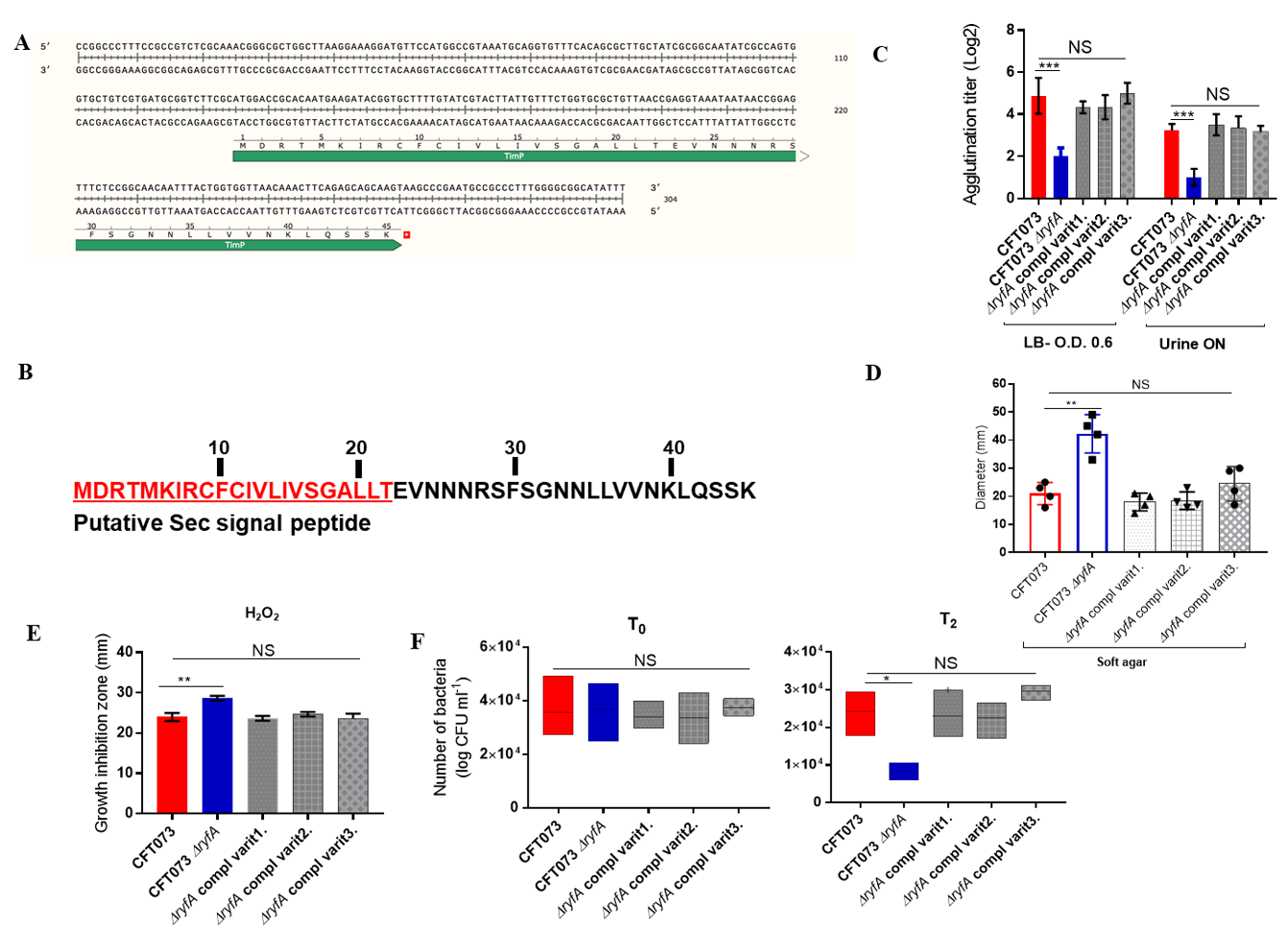

Supplement: S7 Fig — (A) DNA sequence of the ryfA CFT073 predicted ORF corresponding to TimP is indicated with a green arrow. (B) The predicted small protein from RyfA carries a putative Sec system signal sequence in CFT073 (shown in red). (C) Type 1 fimbriae production determined by yeast agglutination in strains cultured to the mid-log phase of growth in LB broth and O/N in urine and (D) Motility in 0.25% soft agar of CT073, isogenic ryfA mutant and the different variant complemented mutants. (E) Growth inhibition zones (mm) of CFT073 and its derivative strains to oxidative stress generating compounds (30% H2O2) on LB agar. (F) THP-1 human macrophages were infected (MOI: 20) with different strains for 1 h, followed by gentamicin treatment. Cells were lysed and intracellular bacterial counts (CFU ml−1) were determined at 2 h p.i. Data represent the averages of at least three separate experiments. Error bars represent the SEM. Statistical significance was calculated by one-way ANOVA: *, P < 0.05; **, P < 0.005; ***, P < 0.0001. NS, not significant. (TIF) [file ppat.1009617.s007.tif]

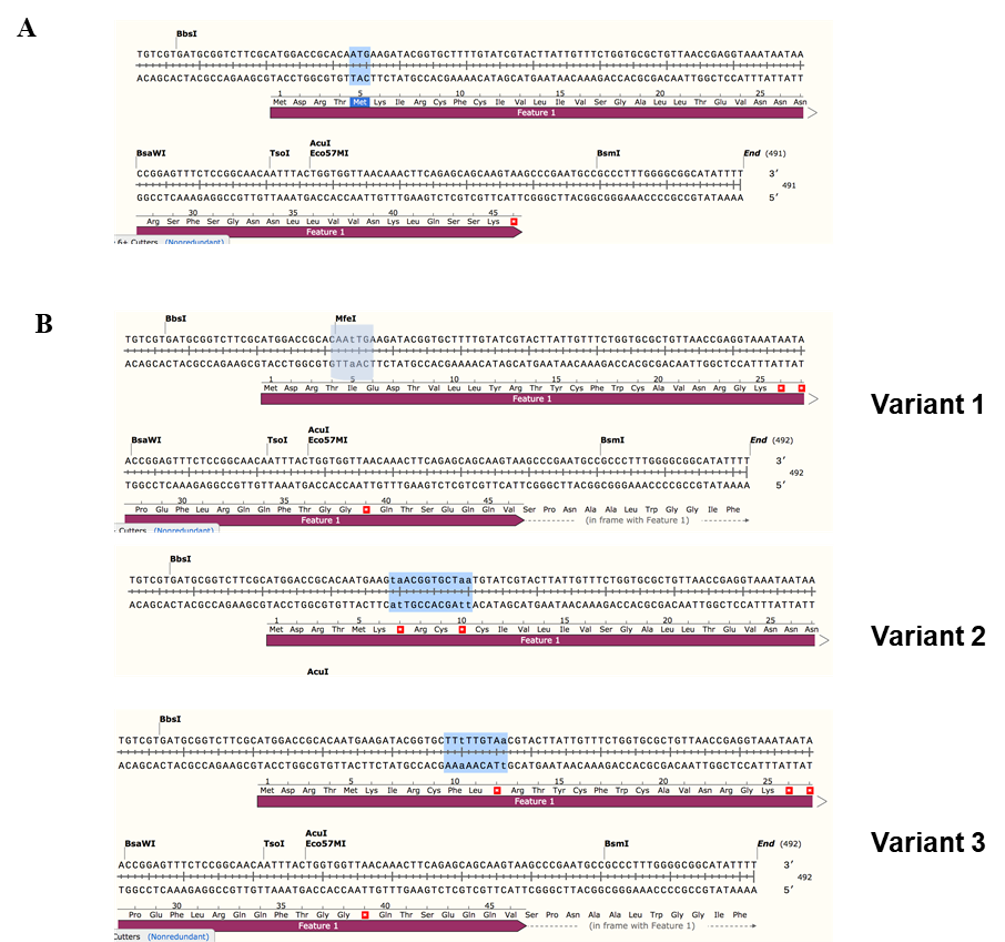

Supplement: S8 Fig — (A) The predicted peptide potentially associated with the RyfA RNA from CFT073. Native ORF present in E. coli CFT073 (B) Variant ryfA alleles wherein small sequence changes would eliminate or alter the ORF through introduction of stop codons or frame-shifts. These three variants of ryfA would not contain the predicted ORF and could not produce such a peptide. (TIF) [file ppat.1009617.s008.tif]

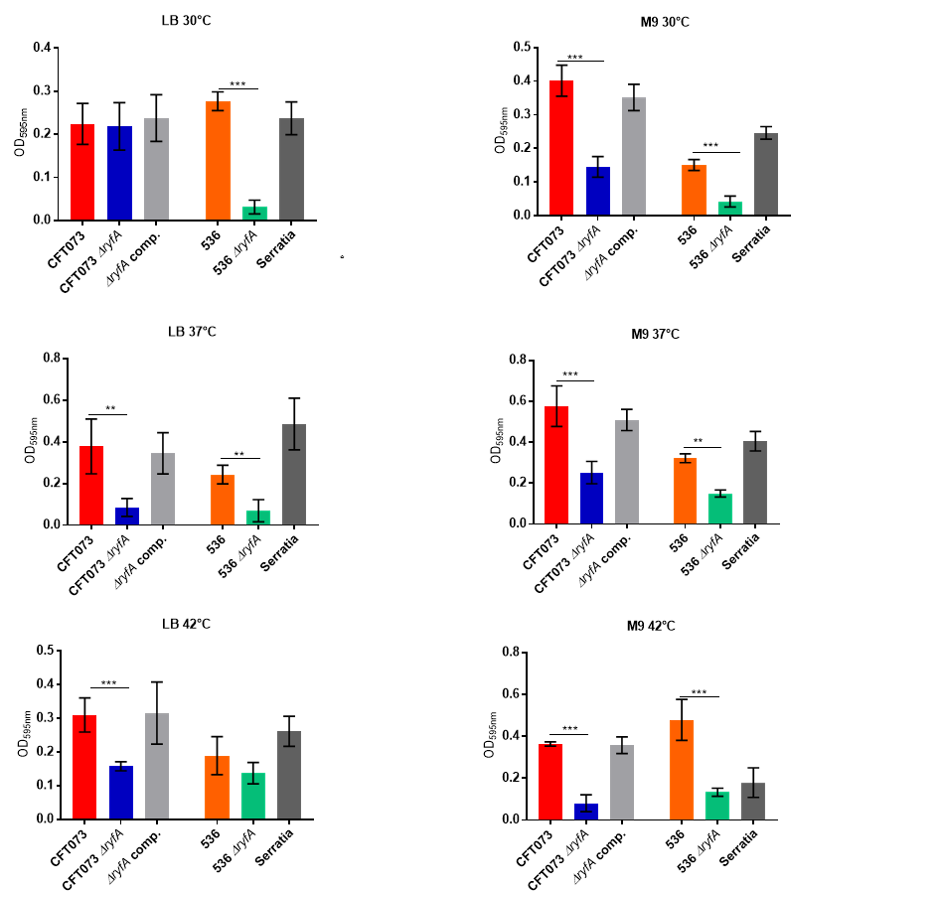

Supplement: S9 Fig — UPEC and Serratia liquefaciens strain were grown at different temperatures (30°C, 37°C, and 42°C) in LB and minimal M9 medium containing 0.2% glucose in polystyrene plate wells for 48 h and then stained with crystal violet. Remaining crystal violet after washing with acetone was measured as absorbance at 595 nm. Data are the means of three independent experiments, and error bars represent standard errors of the means. The Serratia liquefaciens strain was used as positive control for biofilm formation. *, P < 0.05; **, P < 0.005; ***, P < 0.0001 compared to CFT073 or 536 using one-way ANOVA. (TIF) [file ppat.1009617.s009.tif]

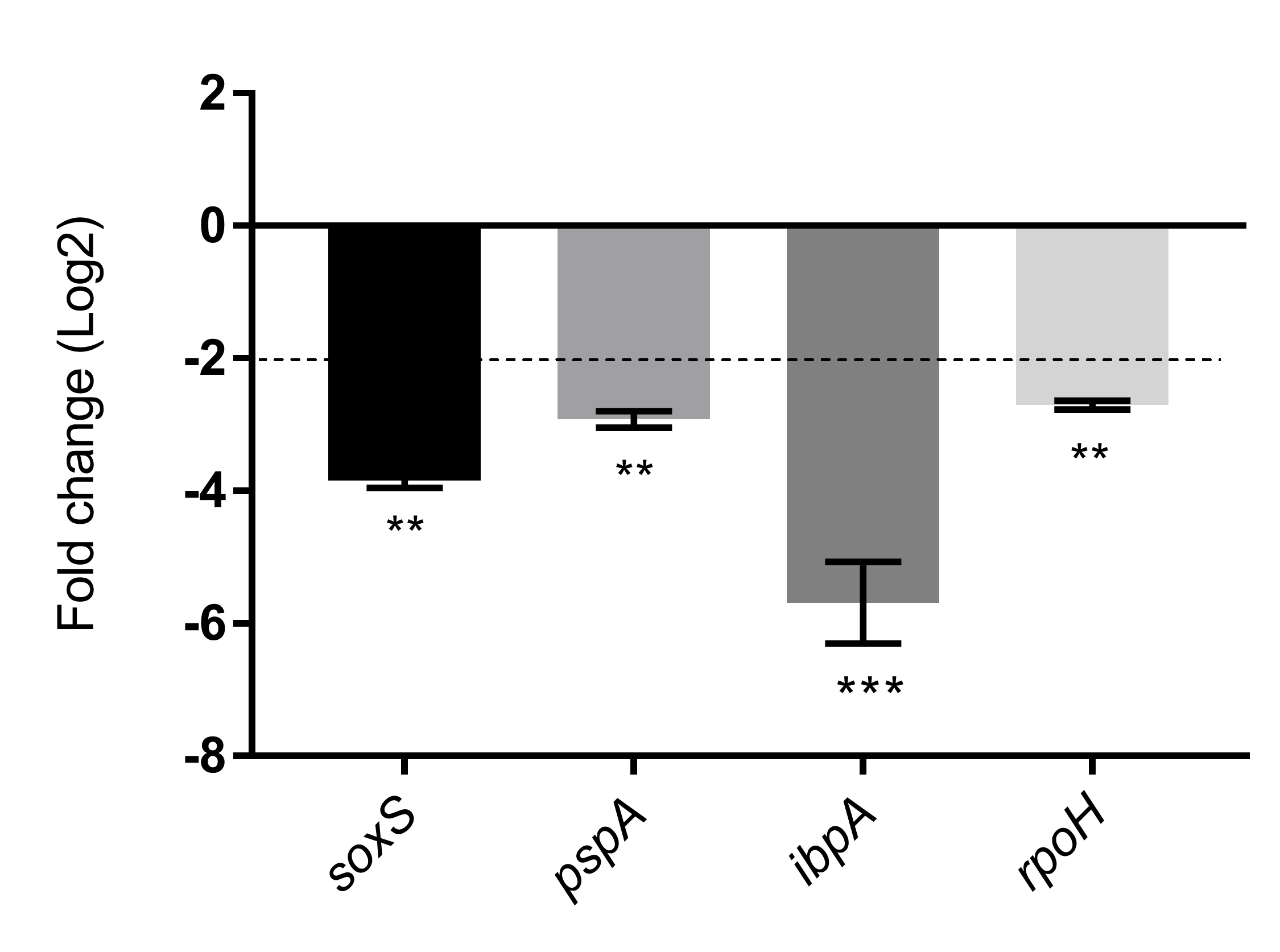

Supplement: S10 Fig — UPEC genes associated with intramacrophage survival. HMDMs were infected at an MOI of 20. Intracellular bacterial survival was assessed at 6 h post-infection and the relative quantity of mRNA of specific genes were determined by qRT-PCR. qPCR data represent means of relative expression ± range (n = 3) of three biological replicates (* p < 0.05, **p < 0.01, ***p < 0.001 using one-way ANOVA). Results were normalized against the steady state RNA levels of rpoD (see Methods for experimental details). (TIF) [file ppat.1009617.s010.tif]
