# Supplementary material for: The RyfA small RNA regulates oxidative and osmotic stress responses and virulence in uropathogenic Escherichia coli
Source: PLoS Pathog. 2021 May 27;17(5):e1009617. doi: 10.1371/journal.ppat.1009617 (PMC8205139; doi:10.1371/journal.ppat.1009617)
Supplement: S1 Table — Strains were inoculated in triplicate 1:100 from an overnight pre-culture grown in LB and grown until mid-log phase with shaking (250 rpm). (PDF) [file ppat.1009617.s011.pdf]

		Upregulated	Downregulated		
Gene Locus	Fold change	Gene symb	CDS	P-value	False Discovery Rate
ibpB	-7,69	*ibpB*	*c4606*	3,5E-06	8,31E-05
C_RS24315	7,61	*cadA*	*c5140*	6,5E-08	8,56E-06
C_RS24320	7,17	*cadB*	*c5141*	5,0E-07	2,37E-05
C_RS00345	7,05	*araA*	*c0074*	8,7E-07	3,30E-05
glpB	6,58	*glpB*	*c2783*	2,2E-10	1,91E-07
C_RS13205	6,56	*glpA*	*c2782*	1,9E-09	1,04E-06
C_RS26455	-6,40			1,6E-05	2,17E-04
C_RS13200	6,19	*glpT*	*c2781*	1,4E-11	7,02E-08
glpC	6,06	*glpC*	*c2784*	2,1E-10	1,91E-07
ibpA	-5,96	*ibpA*	*c4607*	1,2E-06	4,14E-05
C_RS27365	-5,74			1,5E-07	1,41E-05
cspG	-5,65	*cspG*	*c1123*	1,7E-06	5,20E-05
glcD	-5,63	*glcD*	*c3709*	2,0E-06	5,86E-05
C_RS15120	-5,52	*cspI*	*c3177*	2,5E-09	1,12E-06
glcE	-5,47	*glcE*	*c3708*	8,2E-06	1,38E-04
fadB	-5,40	*fadB*	*c4793*	7,2E-10	4,35E-07
treC	5,31	*treC*	*c5338*	1,7E-07	1,46E-05
C_RS13195	5,23	*glpQ*	*c2780*	2,4E-10	1,91E-07
soxS	-5,03	*soxS*	*c5053*	5,8E-11	1,41E-07
C_RS19895	4,97	*glpD*	*c4203*	3,8E-08	6,30E-06
C_RS27330	-4,92		*c3045*	5,5E-06	1,09E-04
C_RS01705	-4,84	*fadE*	*c0371*	5,2E-08	7,74E-06
malE	4,76	*malE*	*c5004*	4,5E-07	2,24E-05
C_RS23715	4,73	*lamB*	*c5006*	2,9E-06	7,54E-05
C_RS23060	4,71	*glpF*	*c4879*	2,8E-10	1,91E-07
C_RS19900	4,65		*c4204*	3,9E-07	2,15E-05
hokA	-4,56	*hokA*		1,1E-06	3,95E-05
kbaY	-4,55	*kbaY*	*c3894*	3,3E-08	5,90E-06
C_RS02185	-4,46		*c0470*	1,5E-07	1,41E-05
glpK	4,41	*glpK*	*c4878*	2,0E-05	2,54E-04
cspA	-4,35	*cspA*	*c3184*	1,9E-06	5,84E-05
C_RS19040	-4,33		*c4016*	3,1E-08	5,90E-06
C_RS12385	-4,32		*c2610*	1,7E-07	1,46E-05
malK	4,29	*malK*	*c5005*	3,5E-06	8,31E-05
C_RS24310	4,10	*dtpC*	*c5139*	2,0E-07	1,52E-05
C_RS14950	-4,07	*ydfK*	*c3145*	1,0E-07	1,09E-05
C_RS22020	-4,04	*asnA*	*c4672*	6,5E-05	5,52E-04
marB	-4,01	*marB*	*c1954*	3,2E-07	2,02E-05
marR	-4,01	*marR*	*c1952*	3,9E-09	1,57E-06
pspG	-4,00	*pspG*	*c5019*	2,0E-08	5,45E-06
C_RS01750	-3,93		*c0381*	9,7E-07	3,64E-05
marA	-3,93	*marA*	*c1953*	8,5E-08	1,00E-05
treB	3,90	*treB*	*c5339*	2,5E-06	6,75E-05
glcC	-3,85	*glcC*	*c3710*	5,9E-07	2,59E-05
C_RS06495	-3,81	*ycfR*	*c1386*	1,3E-07	1,28E-05

bssS	-3,79	*bssS*	*c1327*	1,5E-06	4,85E-05
C_RS12890	-3,78		*c2718*	1,8E-10	1,91E-07
pspB	-3,67	*pspB*	*c1775*	1,1E-08	3,59E-06
C_RS18475	-3,67	*agaS*	*c3893*	8,2E-09	2,85E-06
C_RS25410	-3,66		*c5385*	2,3E-08	5,65E-06
C_RS29385	-3,65			2,3E-07	1,67E-05
C_RS28080	-3,64		*c5169*	1,8E-06	5,54E-05
C_RS06630	-3,63		*c1416*	1,2E-05	1,81E-04
C_RS06580	-3,62	*dicB*		1,4E-05	1,97E-04
azuC	-3,61	*azuC*		1,2E-07	1,19E-05
C_RS25985	-3,60			3,5E-06	8,32E-05
pspC	-3,60	*pspC*	*c1777*	4,0E-08	6,53E-06
nhaA	-3,59	*nhaA*	*c0024*	6,4E-07	2,73E-05
pspD	-3,56	*pspD*	*c1778*	2,4E-07	1,69E-05
C_RS09235	-3,56	*mgtS*		2,2E-07	1,58E-05
C_RS13825	-3,52	*lpxP*	*c2915*	5,8E-07	2,59E-05
C_RS22405	-3,49		*c4741*	3,6E-07	2,11E-05
flgC	3,48	*flgC*	*c1343*	5,9E-03	1,70E-02
ydfZ	3,46	*ydfZ*	*c1967*	1,0E-04	7,59E-04
C_RS10055	-3,46	*ydjM*	*c2127*	7,2E-07	2,92E-05
C_RS09055	-3,40	*safA*	*c1929*	3,8E-04	2,04E-03
C_RS05530	-3,38		*c1176*	7,9E-07	3,13E-05
cpxP	-3,38	*cpxP*	*c4865*	2,5E-05	2,91E-04
C_RS03695	-3,36		*c0785*	2,5E-05	2,91E-04
C_RS25015	-3,32	*exuT*	*c5298*	3,1E-08	5,90E-06
C_RS01320	-3,30		*c0281*	4,4E-07	2,23E-05
agaB	-3,30	*agaB*	*c3895*	1,0E-06	3,69E-05
C_RS26600	-3,29		*c1249*	1,3E-03	5,10E-03
C_RS25430	-3,28	*nanC*	*c5389*	1,2E-06	4,14E-05
C_RS04470	-3,28		*c0943*	2,0E-05	2,54E-04
C_RS12825	-3,27	*fruB*	*c2704*	2,8E-07	1,89E-05
iscR	-3,23	*iscR*	*c3057*	2,5E-09	1,12E-06
C_RS00095	-3,21		*c0021*	4,4E-07	2,24E-05
yrbN	-3,20	*yrbN*		4,2E-06	9,41E-05
C_RS09085	-3,19		*c1935*	9,5E-08	1,07E-05
C_RS17320	-3,18		*c3648*	1,4E-05	1,97E-04
C_RS24510	-3,17	*papC*	*c5186*	3,8E-03	4,89E-03
C_RS13805	-3,16	*yfdX*	*c2911*	8,2E-06	1,38E-04
nikA	3,14	*nikA*	*c4269*	5,9E-07	2,59E-05
C_RS01755	-3,14	*prfH*	*c0382*	3,6E-08	6,23E-06
C_RS15140	-3,12	*essQ*	*c3182*	9,7E-05	7,49E-04
C_RS18260	-3,11	*ygjT*	*c3846*	2,0E-08	5,45E-06
C_RS10360	-3,05	*cdgI*	*c2190*	6,1E-06	1,15E-04
C_RS07455	-3,03		*c1594*	2,3E-05	2,72E-04
nrdH	-3,03	*nrdH*	*c3226*	1,5E-04	9,81E-04
mgtA	-3,03	*mgtA*	*c5341*	3,3E-05	3,46E-04
C_RS03005	-3,02	*ydfM*	*c0649*	3,8E-07	2,15E-05

rrf	-3,02	*rrf*	*c5615*	5,1E-03	1,53E-02
C_RS03010	-3,02	*ybcY*	*c0651*	2,5E-03	8,74E-03
C_RS10705	-3,01	*yebG*	*c2260*	3,6E-07	2,11E-05
C_RS17600	-3,01	*yghO*	*c3711*	4,5E-07	2,25E-05
flgB	3,00	*flgB*	*c1342*	9,3E-03	2,48E-02
C_RS06575	-2,98		*c1404*	2,6E-06	6,96E-05
C_RS20710	-2,98		*c4383*	7,7E-03	2,11E-02
C_RS18450	-2,97	*agaV*	*c3888*	7,1E-08	8,94E-06
C_RS04465	-2,97			1,7E-06	5,41E-05
C_RS18455	-2,93		*c3889*	4,6E-07	2,27E-05
pspA	-2,91	*pspA*	*c1774*	5,0E-06	1,04E-04
glcF	-2,91	*glcF*	*c3707*	5,3E-05	4,84E-04
cspH	-2,90	*cspH*	*c1122*	2,7E-05	3,06E-04
C_RS19660	-2,87	*yhfX*	*c4151*	4,5E-06	9,70E-05
C_RS01315	-2,86		*c0280*	7,2E-08	8,94E-06
C_RS18585	-2,84		*c3916*	4,4E-09	1,64E-06
C_RS12490	-2,82	*rcnA*	*c2633*	2,7E-08	5,90E-06
C_RS21900	-2,82	*bglH*	*c4642*	6,2E-07	2,69E-05
C_RS02510	-2,81	*cyoA*	*c0543*	4,0E-06	9,00E-05
C_RS05910	-2,80		*c5534*	6,9E-06	1,25E-04
C_RS06595	-2,80		*c1409*	1,4E-08	4,20E-06
C_RS08670	-2,79	*ycdI*	*c1847*	4,6E-06	9,86E-05
C_RS17375	-2,79		*c3658*	2,1E-06	6,05E-05
C_RS18445	-2,76	*kbaZ*	*c3887*	1,3E-06	4,31E-05
nikD	2,74	*nikD*	*c4272*	4,4E-07	2,23E-05
dppB	-2,74	*dppB*	*c4358*	3,4E-07	2,09E-05
mntS	-2,73	*mntS*	*c0902*	2,1E-06	6,11E-05
C_RS12075	-2,72	*yefM*	*c2545*	2,2E-08	5,60E-06
C_RS15790	-2,72	*cysJ*	*c3323*	1,0E-07	1,09E-05
nikB	2,71	*nikB*	*c4270*	3,4E-07	2,10E-05
C_RS12845	-2,71	*yeiQ*	*c2709*	1,3E-07	1,28E-05
C_RS12070	-2,70		*c2544*	5,3E-08	7,74E-06
C_RS11985	-2,69		*c2526*	3,9E-06	8,87E-05
uidC	-2,68	*uidC*	*c2007*	1,9E-07	1,52E-05
C_RS01970	-2,68	*ykgH*	*c0425*	8,3E-05	6,67E-04
C_RS05505	-2,68		*c1170*	3,1E-07	2,02E-05
tomB	-2,67	*tomB*	*c0579*	9,9E-06	1,57E-04
C_RS27605	-2,67		*c3653*	4,8E-07	2,31E-05
C_RS29025	-2,66		*c0394*	1,5E-05	2,02E-04
C_RS11545	-2,66		*c2437*	2,5E-04	1,45E-03
glcA	-2,64	*glcA*	*c3704*	1,5E-05	2,05E-04
fxsA	-2,62	*fxsA*	*c5223*	3,0E-05	3,19E-04
C_RS18695	-2,62	*yhbE*	*c3941*	8,7E-07	3,30E-05
C_RS10155	-2,60	*astC*	*c2148*	4,8E-04	2,44E-03
C_RS18050	-2,60		*c3798*	8,1E-06	1,37E-04
C_RS19035	-2,59		*c4015*	4,2E-05	4,09E-04
cysI	-2,59	*cysI*	*c3322*	1,3E-04	8,99E-04

nrdI	-2,58	nrdI	c3227	8,7E-05	6,94E-04
C_RS07160	2,58			7,1E-03	1,99E-02
C_RS03155	-2,58	fepD	c0677	2,7E-04	1,57E-03
pepE	2,57	pepE	c4980	2,0E-05	2,53E-04
C_RS01305	-2,57		c0278	1,6E-06	5,09E-05
C_RS18460	-2,56		c3890	5,2E-08	7,74E-06
C_RS10280	-2,56	ydjE	c2173	7,0E-07	2,89E-05
C_RS16725	-2,55		c3523	2,0E-07	1,52E-05
C_RS18225	-2,55		c3839	6,8E-07	2,86E-05
malM	2,54	malM	c5007	1,5E-04	9,88E-04
C_RS07660	-2,54		c1638	1,5E-04	1,02E-03
C_RS05295	-2,54	ymcE	c1124	1,8E-03	6,61E-03
chaA	-2,52	chaA	c1676	1,3E-07	1,28E-05
C_RS23690	2,51	malG	c5002	2,6E-06	6,90E-05
C_RS28180	-2,50			2,8E-02	5,93E-02
C_RS06010	-2,50		c1280	5,3E-05	4,84E-04
C_RS10200	2,48		c2158	5,8E-08	8,09E-06
C_RS13960	-2,48		c2938	3,8E-03	1,20E-02
C_RS09375	-2,48			2,8E-05	3,09E-04
nikC	2,47	nikC	c4271	4,1E-07	2,22E-05
inaA	2,47	inaA	c2779	5,8E-03	1,68E-02
C_RS08255	-2,46		c1761	5,0E-07	2,37E-05
agaC	-2,46	agaC	c3896	2,1E-06	6,13E-05
C_RS01830	2,45		c0399	4,2E-04	2,19E-03
C_RS12380	-2,45		c2609	1,8E-06	5,54E-05
C_RS08845	-2,44		c1885	2,4E-07	1,69E-05
C_RS29085	-2,43	yliM		7,5E-07	3,00E-05
C_RS23625	-2,43		c4986	1,6E-07	1,46E-05
C_RS03205	-2,42	hcxA	c0687	2,6E-08	5,90E-06
dppC	-2,41	dppC	c4357	1,7E-07	1,48E-05
C_RS24300	2,41	ghoT	c5136	1,5E-06	4,74E-05
C_RS29230	-2,40			5,3E-04	2,59E-03
entS	-2,40	entS	c0678	1,0E-02	2,62E-02
C_RS01310	-2,40		c0279	2,1E-07	1,55E-05
phoE	-2,40	phoE	c0388	1,0E-07	1,09E-05
C_RS15400	-2,40		c3243	3,2E-08	5,90E-06
C_RS03565	-2,40		c0761	4,0E-07	2,19E-05
C_RS20455	2,39		c4329	3,0E-06	7,61E-05
C_RS03760	-2,39	cybA	c0798	6,5E-08	8,56E-06
C_RS24545	-2,39		c5193	9,6E-07	3,59E-05
C_RS28500	-2,37	rzoD		1,8E-04	1,12E-03
C_RS21445	2,36		c4545	8,4E-04	3,71E-03
C_RS21895	-2,35		c4641	5,7E-08	8,09E-06
uxuA	-2,35	uxuA	c5402	1,2E-05	1,75E-04
C_RS04010	-2,35		c0846	1,6E-04	1,05E-03
C_RS28535	-2,35		c1812	5,1E-06	1,04E-04
C_RS13180	2,34	nrdB	c2777	1,1E-05	1,67E-04

C_RS17385	-2,34	tnpB	c3660	1,6E-05	2,17E-04
C_RS00780	-2,33	yadC	c0166	1,2E-05	1,79E-04
malF	2,32	malF	c5003	3,2E-04	1,77E-03
C_RS06320	2,32	flgG	c1347	1,0E-04	7,59E-04
ompW	2,32	ompW	c1722	1,8E-04	1,11E-03
C_RS29075	-2,31			2,3E-07	1,66E-05
flgD	2,31	flgD	c1344	8,3E-03	2,25E-02
C_RS16305	2,31	lysA	c3435	5,3E-05	4,81E-04
astD	-2,31	astD	c2146	2,3E-03	8,12E-03
C_RS17515	2,31		c3694	2,1E-06	6,13E-05
C_RS04475	-2,31		c0944	2,2E-06	6,25E-05
C_RS28530	-2,30		c1811	7,7E-04	3,46E-03
proV	-2,30	proV	c3230	8,1E-05	6,58E-04
C_RS11445	-2,30		c2415	1,3E-03	5,09E-03
cysA	-2,29	cysA	c2956	2,6E-06	6,96E-05
C_RS07625	-2,29		c1630	3,4E-06	8,25E-05
htpX	-2,28	htpX	c2238	1,0E-06	3,69E-05
cysT	-2,28	cysT	c2958	2,4E-06	6,60E-05
yhcN	-2,28	yhcN	c3993	8,3E-06	1,39E-04
C_RS28455	-2,28		c1421	1,6E-06	4,96E-05
C_RS24230	2,28		c5121	1,9E-05	2,45E-04
C_RS11355	-2,28		c2396	1,6E-03	6,24E-03
C_RS02175	-2,27		c0468	1,6E-04	1,03E-03
C_RS15430	-2,26		c5561	1,8E-02	4,21E-02
C_RS27370	2,26			1,2E-04	8,67E-04
C_RS25005	-2,26		c5295	1,3E-06	4,38E-05
dhaL	2,25	dhaL	c1657	3,8E-06	8,86E-05
C_RS17410	-2,25		c3668	8,2E-06	1,38E-04
C_RS15765	-2,25	cysD	c3319	4,7E-05	4,44E-04
gntP	-2,24	gntP	c5401	1,0E-05	1,63E-04
C_RS09685	-2,24		c2051	4,7E-03	1,44E-02
C_RS14780	-2,24	kgtP	c3112	2,1E-06	6,05E-05
ilvB	-2,24	ilvB	c4596	1,4E-05	1,93E-04
fadI	-2,24	fadI	c2887	5,5E-06	1,09E-04
C_RS02025	-2,23		c0435	1,4E-06	4,50E-05
C_RS08390	-2,23	ompG	c1791	3,9E-05	3,89E-04
C_RS13765	-2,22	emrY	c2902	6,5E-05	5,57E-04
C_RS11450	-2,22			1,4E-03	5,37E-03
C_RS12820	-2,21	fruK	c2703	4,0E-06	9,00E-05
trxC	-2,21	trxC	c3107	2,1E-05	2,60E-04
C_RS10530	2,21		c2226	1,5E-03	5,89E-03
C_RS06215	-2,20		c1323	8,1E-06	1,37E-04
higA	-2,20	higA	c3840	6,0E-06	1,15E-04
dnaJ	-2,20	dnaJ	c0020	3,6E-04	1,95E-03
C_RS04255	-2,20		c0901	2,1E-05	2,59E-04
C_RS06220	-2,20		c1325	1,1E-05	1,72E-04
C_RS11790	-2,20		c2489	8,7E-04	3,80E-03

C_RS20705	-2,20		c4382	3,1E-03	1,02E-02
C_RS10150	-2,20	astA	c2147	1,3E-02	3,18E-02
C_RS07240	-2,19		c1548	8,9E-06	1,46E-04
C_RS03045	-2,19	cusC	c0658	3,0E-05	3,19E-04
C_RS13055	-2,19	yojI	c2752	1,1E-02	2,93E-02
C_RS24260	2,19		c5127	7,0E-07	2,89E-05
C_RS19230	-2,19		c4052	1,5E-04	1,02E-03
C_RS07280	2,19			1,4E-02	3,53E-02
nrdD	2,19	nrdD	c5337	8,0E-08	9,67E-06
yfdV	-2,18	yfdV		5,6E-06	1,10E-04
fliN	2,18	fliN	c2363	2,0E-02	4,51E-02
lysS	2,17	lysS	c3469	3,0E-07	1,97E-05
C_RS17575	-2,17		c3706	4,3E-04	2,21E-03
C_RS20590	-2,17	dppD	c4356	6,9E-06	1,25E-04
C_RS21420	-2,17		c4537	1,8E-07	1,49E-05
C_RS14640	-2,17	shoB		3,6E-03	1,16E-02
C_RS23020	2,17	tpiA	c4871	4,7E-03	1,44E-02
C_RS08940	2,17			3,7E-05	3,76E-04
C_RS03820	2,16		c0812	7,4E-03	2,04E-02
hspQ	-2,16	hspQ	c1104	2,7E-04	1,55E-03
cydX	2,16	cydX		7,1E-07	2,90E-05
hycA	2,16	hycA	c3285	3,8E-02	7,60E-02
lysC	2,16	lysC	c4990	4,3E-07	2,23E-05
C_RS11435	-2,16		c2413	1,3E-04	8,97E-04
C_RS24265	2,15		c5128	5,9E-06	1,13E-04
C_RS11370	2,15		c2399	5,2E-05	4,80E-04
C_RS11125	2,15		c2348	1,1E-04	8,13E-04
recX	-2,15	recX	c3252	2,1E-06	6,05E-05
C_RS23905	-2,14		c5050	4,2E-07	2,22E-05
C_RS10700	-2,14	yebF	c2259	8,2E-03	2,23E-02
C_RS17815	-2,14		c3751	5,1E-05	4,74E-04
C_RS05705	-2,14		c1214	1,1E-05	1,67E-04
C_RS14060	-2,13		c2959	4,2E-07	2,22E-05
C_RS09045	2,13		c1927	7,9E-06	1,35E-04
C_RS11990	-2,13		c2527	1,7E-04	1,07E-03
gcvH	2,13	gcvH	c3484	1,2E-05	1,80E-04
C_RS10835	-2,12		c2287	1,8E-04	1,11E-03
C_RS24390	-2,12		c5156	3,5E-05	3,57E-04
C_RS28815	-2,12		c3674	3,3E-06	8,25E-05
C_RS05510	-2,11		c1171	3,2E-05	3,36E-04
C_RS09090	-2,11		c1936	4,0E-04	2,12E-03
C_RS25590	2,11		c5418	5,4E-05	4,86E-04
metR	-2,11	metR	c4750	3,6E-07	2,11E-05
C_RS25720	-2,11		c5444	4,8E-06	1,02E-04
C_RS23620	-2,11		c4985	2,7E-05	3,03E-04
C_RS07330	-2,11	iss	c1564	3,8E-03	1,21E-02
glcB	-2,10	glcB	c3705	1,9E-04	1,17E-03

C_RS07550	-2,10		c1612	5,2E-05	4,80E-04
C_RS10170	2,10	ydjY	c2152	1,0E-06	3,69E-05
C_RS02655	-2,09	hha	c0578	7,6E-05	6,23E-04
C_RS28410	-2,09		c1258	5,0E-07	2,37E-05
dhaK	2,09	dhaK	c1658	2,6E-04	1,49E-03
C_RS01650	-2,08		c0360	4,3E-05	4,15E-04
umuD	-2,08	umuD	c1631	9,4E-05	7,36E-04
cydA	2,07	cydA	c0811	5,7E-05	5,05E-04
C_RS13175	2,07	nrdA	c2776	1,2E-05	1,78E-04
C_RS13355	-2,07			1,0E-04	7,84E-04
betI	-2,06	betI	c0433	4,1E-04	2,16E-03
C_RS13955	-2,06	xapR	c2937	4,9E-06	1,02E-04
C_RS06020	-2,06			1,6E-03	6,13E-03
C_RS06585	-2,06			5,4E-06	1,07E-04
nhaR	-2,06	nhaR	c0025	1,7E-07	1,48E-05
C_RS06310	2,06	flgE	c1345	7,6E-04	3,43E-03
C_RS02490	-2,05	cyoE	c0539	3,6E-04	1,95E-03
C_RS17525	2,05		c3696	1,3E-04	9,21E-04
C_RS29405	-2,05			7,1E-03	1,99E-02
C_RS09080	-2,05		c1934	7,1E-06	1,26E-04
C_RS01600	-2,05			4,8E-03	1,45E-02
C_RS13895	-2,05		c2931	5,9E-04	2,82E-03
C_RS15290	-2,04	ygaV	c3216	1,1E-04	8,13E-04
C_RS29450	-2,04			1,0E-06	3,69E-05
C_RS14565	-2,04		c3070	1,4E-04	9,71E-04
C_RS17510	2,04		c3693	2,3E-05	2,74E-04
pyrB	2,04	pyrB	c5345	6,5E-06	1,20E-04
C_RS25190	2,04	nrdG	c5336	1,8E-07	1,49E-05
grcA	2,04	grcA	c3103	3,8E-03	1,21E-02
C_RS05525	-2,04		c1175	4,2E-07	2,22E-05
C_RS14050	-2,03	cysW	c2957	2,1E-06	6,13E-05
C_RS01400	-2,03			2,8E-03	9,52E-03
C_RS15880	2,03	eno	c3344	4,8E-03	1,46E-02
C_RS17030	-2,03	papC	c3590	5,1E-02	6,11E-01
C_RS04585	2,02		c0970	2,1E-04	1,25E-03
C_RS19855	2,02	malP	c4194	3,0E-03	1,01E-02
dnaK	-2,02	dnaK	c0019	4,6E-04	2,35E-03
recA	-2,01	recA	c3253	6,6E-03	1,88E-02
dinD	-2,01	dinD	c4469	1,1E-06	4,04E-05
C_RS11340	2,01		c2393	2,7E-03	9,25E-03
C_RS15920	-2,00	gudP	c3353	1,3E-02	3,29E-02
C_RS19665	-2,00		c4152	4,4E-04	2,25E-03
C_RS02505	-2,00	cyoB	c0542	7,5E-05	6,21E-04
C_RS18370	2,00		c3871	1,5E-02	3,56E-02
C_RS06625	-2,00		c1414	3,9E-04	2,06E-03
C_RS28095	-2,00			2,7E-02	5,83E-02
C_RS28025	-1,99			1,6E-05	2,17E-04

C_RS14445	-1,99	ndk	c3041	5,1E-04	2,54E-03
C_RS17520	1,99		c3695	3,6E-06	8,51E-05
C_RS09070	-1,99		c1932	3,1E-05	3,29E-04
C_RS15560	-1,99		c3274	2,8E-04	1,58E-03
C_RS18420	-1,99	garP	c3882	1,3E-02	3,28E-02
C_RS01880	-1,99			1,7E-03	6,31E-03
C_RS17705	1,98	hybB	c3732	7,2E-03	2,01E-02
C_RS09215	-1,97		c1959	1,6E-03	6,13E-03
tkt	1,97	tkt	c2990	5,5E-04	2,68E-03
C_RS05790	-1,97			6,2E-05	5,36E-04
cspF	-1,97	cspF	c3185	3,0E-06	7,76E-05
C_RS17690	1,97	hybE	c3729	1,6E-04	1,02E-03
C_RS02885	-1,97		c0625	2,4E-05	2,84E-04
sulA	-1,96	sulA	c1095	2,6E-05	2,98E-04
C_RS17700	1,96	hybC	c3731	4,9E-06	1,02E-04
C_RS13785	-1,96	yfdE	c2907	2,3E-06	6,55E-05
C_RS13185	1,96	yfaE	c2778	1,1E-05	1,74E-04
C_RS12810	1,96		c2701	6,9E-03	1,96E-02
nikE	1,96	nikE	c4273	9,2E-06	1,50E-04
C_RS24060	-1,96		c5088	1,4E-03	5,52E-03
ftnA	1,96	ftnA	c2321	2,3E-03	8,08E-03
C_RS16650	1,96		c3506	2,5E-04	1,47E-03
C_RS18465	-1,96		c3891	2,2E-03	7,72E-03
C_RS14945	-1,95		c3144	1,8E-06	5,51E-05
C_RS09210	-1,95		c1958	2,0E-04	1,23E-03
pstS	-1,94	pstS	c4653	5,0E-05	4,67E-04
proW	-1,94	proW	c3231	5,9E-05	5,21E-04
C_RS06315	1,94	flgF	c1346	5,0E-04	2,50E-03
C_RS04015	-1,94		c0847	4,3E-05	4,15E-04
C_RS10525	1,93	manY	c2224	2,2E-04	1,33E-03
C_RS05500	-1,93			5,2E-05	4,80E-04
C_RS16825	1,93	ansB	c3543	6,3E-02	1,16E-01
tnaB	-1,92	tnaB	c4632	1,2E-02	3,00E-02
C_RS07215	-1,92			3,0E-04	1,69E-03
C_RS11375	1,92		c2400	1,4E-04	9,48E-04
C_RS07515	-1,92	bluF	c1606	1,2E-06	4,14E-05
C_RS20265	1,92		c4285	1,4E-04	9,47E-04
C_RS07870	1,91	narG	c1685	2,0E-05	2,50E-04
C_RS11855	-1,91		c2500	2,6E-04	1,50E-03
C_RS25060	-1,91	cycA	c5307	4,4E-06	9,66E-05
cheW	1,91	cheW	c2302	2,7E-02	5,75E-02
C_RS02635	-1,90		c0574	1,4E-03	5,67E-03
C_RS24295	1,90	ghoS		3,2E-06	8,04E-05
C_RS14955	-1,90		c3146	5,4E-06	1,08E-04
C_RS02500	-1,90	cyoC	c0541	2,7E-04	1,55E-03
C_RS14035	-1,89		c2954	1,4E-06	4,55E-05
C_RS09245	-1,89	dgcZ	c1963	9,2E-08	1,06E-05

C_RS10335	1,89		c2185	1,5E-04	1,02E-03
C_RS02650	-1,89	maa	c0577	6,1E-04	2,90E-03
ypdK	-1,89	ypdK		5,7E-04	2,74E-03
C_RS15080	-1,88		c3172	6,3E-02	1,15E-01
C_RS11700	-1,88	clbC	c2468	5,6E-04	2,72E-03
prpB	-1,88	prpB	c0451	3,9E-06	8,90E-05
C_RS03440	-1,88	gltI	c0739	1,5E-04	9,88E-04
C_RS02495	-1,88		c0540	9,7E-04	4,13E-03
fliJ	1,88	fliJ	c2359	8,7E-03	2,35E-02
C_RS09035	-1,87		c1925	7,1E-05	5,96E-04
C_RS15555	-1,87		c3273	1,3E-03	5,09E-03
C_RS06025	-1,87			3,0E-03	1,01E-02
C_RS15425	-1,87		c5560	7,1E-05	5,95E-04
C_RS08615	-1,87		c1835	3,9E-06	8,91E-05
focA	1,87	focA	c1239	2,0E-07	1,52E-05
ghrB	1,86	ghrB	c4372	4,0E-05	3,97E-04
C_RS10420	-1,86		c2202	1,3E-04	9,22E-04
C_RS17235	-1,85		c3628	3,5E-07	2,11E-05
C_RS01295	-1,85		c0275	8,9E-04	3,86E-03
nagA	-1,85	nagA	c0752	3,3E-05	3,46E-04
fbaA	1,85	fbaA	c3503	3,3E-05	3,40E-04
C_RS17695	1,84		c3730	1,4E-05	2,01E-04
C_RS01460	-1,84		c0317	2,2E-03	7,78E-03
C_RS08830	-1,83		c1882	1,1E-04	8,13E-04
nac	-1,83	nac	c2446	7,1E-06	1,26E-04
C_RS03435	-1,83	gltJ	c0738	2,3E-06	6,38E-05
hybA	1,83	hybA	c3733	1,1E-04	8,13E-04
C_RS14505	-1,82	iscS	cysteine des	5,4E-03	1,59E-02
C_RS18355	-1,82		c3867	3,2E-05	3,36E-04
C_RS08400	-1,82		c1793	3,2E-05	3,36E-04
C_RS08735	-1,82		c1860	7,0E-06	1,26E-04
C_RS25965	-1,82			5,0E-02	9,57E-02
C_RS20120	-1,82	livJ	c4253	5,0E-06	1,04E-04
C_RS09075	-1,81		c1933	3,3E-06	8,25E-05
fadA	-1,81	fadA	c4792	8,2E-07	3,21E-05
C_RS17785	1,81	yqhD	c3745	2,7E-03	9,17E-03
C_RS17505	1,81		c3692	1,0E-04	7,75E-04
C_RS23615	-1,81		c4984	2,8E-05	3,10E-04
aceE	1,81	aceE	c0142	1,5E-04	9,73E-04
argO	-1,80	argO	c3501	2,0E-05	2,49E-04
C_RS09250	-1,80			5,9E-07	2,59E-05
C_RS00100	-1,80		c0022	2,1E-05	2,60E-04
dapD	1,80	dapD	c0201	4,3E-04	2,22E-03
malI	-1,80	malI	c2012	7,0E-05	5,90E-04
C_RS05765	-1,80		c1231	3,1E-04	1,72E-03
fruA	-1,80	fruA	c2702	2,3E-02	5,06E-02
C_RS17240	-1,80		c3630	3,6E-07	2,11E-05

C_RS05515	-1,80		*c1173*	6,9E-06	1,25E-04
C_RS01800	-1,79		*c0391*	8,4E-07	3,24E-05
garL	-1,79	*garL*	*c3881*	2,3E-02	5,21E-02
xapA	-1,79	*xapA*	*c2940*	3,8E-03	1,21E-02
C_RS14295	1,79	*bcp*	*c3008*	9,3E-04	4,01E-03
C_RS22920	-1,78		*c4849*	6,4E-07	2,73E-05
C_RS19755	-1,78	*hslR*	*c4171*	2,2E-05	2,66E-04
C_RS17405	-1,78		*c3666*	1,2E-02	3,00E-02
C_RS00685	-1,78		*c0146*	1,4E-06	4,58E-05
C_RS10285	-1,78		*c2174*	4,3E-04	2,22E-03
C_RS16335	-1,77		*c3441*	8,5E-05	6,86E-04
fliR	-1,77	*fliR*	*c2367*	1,4E-04	9,73E-04
C_RS01325	-1,77		*c0282*	2,4E-05	2,84E-04
C_RS04485	1,77		*c0946*	1,2E-04	8,64E-04
C_RS04580	1,77		*c0969*	7,8E-04	3,50E-03
C_RS03355	-1,77		*c0721*	7,5E-04	3,41E-03
cysH	-1,77	*cysH*	*c3321*	4,0E-05	3,99E-04
rpoH	-1,77	*rpoH*	*c4254*	5,2E-05	4,76E-04
C_RS13795	-1,77	*oxc*	*c2909*	2,4E-06	6,73E-05
C_RS25225	1,77	*ridA*	*c5342*	1,7E-03	6,41E-03
C_RS29330	-1,77			7,0E-05	5,90E-04
C_RS10365	-1,76		*c2191*	1,4E-05	1,98E-04
C_RS26640	-1,76			3,9E-05	3,89E-04
csgB	-1,76	*csgB*	*c1305*	8,6E-03	2,32E-02
C_RS29105	-1,76			7,3E-06	1,29E-04
C_RS25365	1,75		*c5372*	3,4E-05	3,53E-04
gcvT	1,75	*gcvT*	*c3485*	3,6E-05	3,60E-04
C_RS21255	-1,75		*c4502*	1,9E-07	1,52E-05
C_RS18210	-1,74		*c3836*	9,5E-05	7,40E-04
C_RS01605	-1,74		*c0348*	5,1E-05	4,70E-04
C_RS13950	-1,74		*c5558*	1,9E-02	4,44E-02
garD	-1,74	*garD*	*c3883*	1,7E-02	3,95E-02
C_RS11075	1,74	*rpoF*	*c2337*	4,2E-02	1,60E-01
C_RS01440	-1,73		*c0310*	7,8E-03	2,15E-02
fadD	-1,73	*fadD*	*c2209*	1,6E-05	2,17E-04
C_RS04480	-1,73		*c0945*	7,4E-05	6,12E-04
C_RS23650	1,73	*pgi*	*c4991*	3,0E-05	3,20E-04
ybgE	1,73	*ybgE*	*c0814*	2,8E-07	1,89E-05
serC	1,73	*serC*	*c1045*	7,5E-07	3,00E-05
C_RS27750	-1,73			2,7E-03	9,25E-03
C_RS06050	-1,73		*c1288*	1,8E-03	6,57E-03
C_RS01425	-1,72		*c0307*	7,4E-06	1,30E-04
C_RS19460	-1,72	*bfd*	*c4108*	2,1E-03	7,64E-03
dinI	-1,72	*dinI*	*c1328*	1,2E-03	4,76E-03
mdtI	-1,72	*mdtI*	*c1991*	1,2E-02	3,11E-02
uspC	1,72	*uspC*	*c2309*	1,0E-03	4,37E-03
fliF	1,72	*fliF*	*c2354*	2,9E-02	6,21E-02

C_RS02130	-1,72	*lacA*	*c0457*	8,8E-02	1,52E-01
C_RS10875	1,72	*cheY*	*c2297*	2,8E-03	9,52E-03
C_RS23955	-1,72		*c5063*	3,5E-06	8,35E-05
C_RS26550	-1,71			4,2E-02	8,33E-02
mdtJ	-1,71	*mdtJ*	*c1992*	1,2E-02	2,97E-02
C_RS02180	-1,71		*c0469*	2,1E-05	2,56E-04
fhuF	-1,71	*fhuF*	*c5446*	1,9E-02	4,38E-02
C_RS17180	-1,71	*tnpB*	*c3616*	3,7E-05	3,69E-04
C_RS29220	-1,71		*c1892*	1,6E-02	3,75E-02
C_RS21545	-1,71		*c4571*	9,4E-06	1,53E-04
garK	-1,71	*garK*	*c3879*	6,4E-02	1,17E-01
pepT	1,71	*pepT*	*c1479*	1,2E-05	1,76E-04
C_RS24700	1,71		*c5228*	9,1E-06	1,48E-04
ffs	-1,70	*ffs*		6,2E-05	5,38E-04
C_RS17315	-1,70		*c3647*	6,1E-04	2,91E-03
norR	-1,70	*norR*	*c3263*	9,7E-05	7,46E-04
