# Supplementary material for: The RyfA small RNA regulates oxidative and osmotic stress responses and virulence in uropathogenic Escherichia coli
Source: PLoS Pathog. 2021 May 27;17(5):e1009617. doi: 10.1371/journal.ppat.1009617 (PMC8205139; doi:10.1371/journal.ppat.1009617)
Supplement: S2 Table — (DOCX) [file ppat.1009617.s012.docx]

S2 TABLE: Strains and plasmids used in this study.

| **Strain/plasmid** | **Relevant characteristics** | **Reference or source** |
| --- | --- | --- |
| **Strains** | | |
| CFT073 | UPEC wild-type pyelonephritis strain (O6:K2:H1) | [1,2] |
| 536 | UPEC wild-type pyelonephritis strain  (O6:K15:H31) | [3] |
| QT1324 | CFT073 ΔoxyR::Km; Km^r^ | [4] |
| QT2138 | CFT073 ΔfimAICDFGH::km; Km^r^ | [4] |
| QT2081 | CFT073 ΔlacZYA::FRT | [5] |
| QT2285 | CFT073 fimS phase L-ON, Cm^r^ | [4] |
| QT2496 | CFT073 + pSTNSK, Km^r^ | [4] |
| QT2799 | *Serratia liquefaciens* | ATCC 27592 |
| QT2909 | CFT073 *araBAD::Gm* - pNM12 | This study |
| QT3586 | UTI89 ΔfimH::km; Km^r^ | This study |
| QT4437 | MGN-617 + pGP-Tn7-Cm-Ptrc-mCherryST | This study |
| QT5255 | CFT073 Δ*ryfA::FRT* | This study |
| QT5295 | CFT073, fimS phase L-ON, Δ*ryfA* ::Km; Km^r^ Cm^r^ | This study |
| QT5305 | MGN-617 + pGP-Tn7-ryfA | This study |
| QT5308 | QT5255 + pSTNSK, Km^r^ | This study |
| QT5309 | QT5308 :Tn7T-Cm::*ryfA,*  Cm^r^ | This study |
| QT5325 | QT5255 *araBAD::Gm,*  Cm^r^ | This study |
| QT5399 | QT5325-pNM12, Ap^r^ | This study |
| QT5404 | QT5255 + pKEN2, Ap^r^ | This study |
| QT5559 | QT2496 :Tn7T-Cm::p*trc-mCherryST ,*  Cm^r^ | This study |
| QT5560 | QT5308 :Tn7T-Cm::p*trc-mCherryST ,*  Cm^r^ | This study |
| QT5613 | 536 *ΔryfA::Km,*  Km^r^ | This study |
| QT5656 | CFT073 + pKEN2, Ap^r^ | This study |
| QT5657 | QT5309 + pKEN2, Ap^r^ | This study |
| QT5682 | QT5295 + pKEN2, Ap^r^, Km^r^, Cm^r^ | This study |
| QT5685 | UTI89 *ΔryfA::FRT* | This study |
| QT5708 | QT5685 + pSTNSK, Km^r^ | This study |
| QT5709 | QT5709 :Tn7T-Cm::*ryfA,*  Cm^r^ | This study |
| QT5710 | UTI89 + pKEN2, Ap^r^ | This study |
| QT5711 | QT5685 + pKEN2, Ap^r^ | This study |
| QT5712 | QT3586 + pKEN2, Ap^r^ | This study |
| QT5733 | QT5308 :Tn7T-Cm::*ryfA* variant 1*,*  Cm^r^ | This study |
| QT5734 | QT5308 :Tn7T-Cm::*ryfA* variant 2*,*  Cm^r^ | This study |
| QT5734 | QT5308 :Tn7T-Cm::*ryfA* variant 3*,*  Cm^r^ | This study |
| UTI89 | UPEC wild-type cystitis  strain | [6,7] |
| χ7213 (MGN-617) | *thi thr leu tonA lacY glnV supE ΔasdA4 recA*::RP4 2-Tc::Mu [λpir], Km^r^ | [8] |
| **Plasmids** | | |
| pCP20 | FLP helper plasmid Ts replicon; Ap^r^ Cm^r^ | [9] |
| pGP-Tn7-Cm | pGP-Tn7-FRT:: Cm, Ap^r^, Cm^r^ | [10] |
| pIJ258 (pSTNSK) | pST76-K::*tnsABCD*, Km^r^ | [10] |
| pIJ360 (pSTNSK) | pST76-K::*tnsABCD*, Tp^r^ | [10] |
| pIJ375- mCherryST | pGP-Tn7-Cm-Ptrc-mCherryST | [11] |
| pIJ546 | pGP-Tn7-Cm::*ryfA*; Ap^r^,Cm^r^ | This study |
| pIJ588 | pGP-Tn7-Cm::*ryfA* variant 1 ; Ap^r^,Cm^r^ | This study |
| pIJ589 | pGP-Tn7-Cm::*ryfA* variant 2 ; Ap^r^,Cm^r^ | This study |
| pIJ590 | pGP-Tn7-Cm::*ryfA* variant 3 ; Ap^r^,Cm^r^ | This study |
| pKD3 | Template plasmid for the amplification of the cat gene bordered by FRT sites | [9] |
| pKD4 | Template plasmid for the amplification of the km cassette bordered by FRT sites | [9] |
| pKD46 | λ-Red recombinase plasmid Ts replicon; Ap^r^ | [9] |
| pKEN2 | high copy phagemid constitutively expressing GFP, Ap^r^ | [12] |
| pNM12 | pBAD24 derivative, Ap^r^ | [13] |

**References**

1. Mobley H, Green D, Trifillis A, Johnson D, Chippendale G, Lockatell C, et al. Pyelonephritogenic *Escherichia coli* and killing of cultured human renal proximal tubular epithelial cells: role of hemolysin in some strains. Infection and immunity. 1990;58(5):1281-9.

2. Welch RA, Burland V, Plunkett G, 3rd, Redford P, Roesch P, Rasko D, et al. Extensive mosaic structure revealed by the complete genome sequence of uropathogenic *Escherichia coli*. Proc Natl Acad Sci U S A. 2002;99(26):17020-4. Epub 2002/12/10. doi: 10.1073/pnas.252529799. PubMed PMID: 12471157; PubMed Central PMCID: PMC139262.

3. Berger H, Hacker J, Juarez A, Hughes C, Goebel W. Cloning of the chromosomal determinants encoding hemolysin production and mannose-resistant hemagglutination in *Escherichia coli*. Journal of bacteriology. 1982;152(3):1241-7.

4. Crepin S, Houle S, Charbonneau ME, Mourez M, Harel J, Dozois CM. Decreased expression of type 1 fimbriae by a pst mutant of uropathogenic *Escherichia coli* reduces urinary tract infection. Infect Immun. 2012;80(8):2802-15. Epub 2012/06/06. doi: 10.1128/iai.00162-12. PubMed PMID: 22665376; PubMed Central PMCID: PMC3434566.

5. Sabri M, Houle S, Dozois CM. Roles of the extraintestinal pathogenic *Escherichia coli* ZnuACB and ZupT zinc transporters during urinary tract infection. Infection and immunity. 2009;77(3):1155-64.

6. Mulvey MA, Schilling JD, Hultgren SJ. Establishment of a persistent *Escherichia coli* reservoir during the acute phase of a bladder infection. Infection and immunity. 2001;69(7):4572-9.

7. Chen SL, Hung C-S, Xu J, Reigstad CS, Magrini V, Sabo A, et al. Identification of genes subject to positive selection in uropathogenic strains of *Escherichia coli*: a comparative genomics approach. Proceedings of the National Academy of Sciences. 2006;103(15):5977-82.

8. Kaniga K, Compton MS, Curtiss R, 3rd, Sundaram P. Molecular and functional characterization of *Salmonella enterica* serovar *typhimurium* *poxA* gene: effect on attenuation of virulence and protection. Infect Immun. 1998;66(12):5599-606. Epub 1998/11/24. PubMed PMID: 9826331; PubMed Central PMCID: PMC108707.

9. Datsenko KA, Wanner BL. One-step inactivation of chromosomal genes in *Escherichia coli* K-12 using PCR products. Proceedings of the National Academy of Sciences. 2000;97(12):6640-5.

10. Crépin S, Harel J, Dozois CM. Chromosomal complementation using Tn7 transposon vectors in Enterobacteriaceae. Appl Environ Microbiol. 2012;78(17):6001-8.

11. Knodler LA, Crowley SM, Sham HP, Yang H, Wrande M, Ma C, et al. Noncanonical inflammasome activation of caspase-4/caspase-11 mediates epithelial defenses against enteric bacterial pathogens. Cell host & microbe. 2014;16(2):249-56.

12. Cormack BP, Valdivia RH, Falkow S. FACS-optimized mutants of the green fluorescent protein (GFP). Gene. 1996;173(1):33-8.

13. Majdalani N, Cunning C, Sledjeski D, Elliott T, Gottesman S. DsrA RNA regulates translation of RpoS message by an anti-antisense mechanism, independent of its action as an antisilencer of transcription. Proceedings of the National Academy of Sciences. 1998;95(21):12462-7.
