# Supplementary material for: The RyfA small RNA regulates oxidative and osmotic stress responses and virulence in uropathogenic Escherichia coli
Source: PLoS Pathog. 2021 May 27;17(5):e1009617. doi: 10.1371/journal.ppat.1009617 (PMC8205139; doi:10.1371/journal.ppat.1009617)
Supplement: S3 Table — (PDF) [file ppat.1009617.s013.pdf]

Primers	Direction	Characteristic (s)	Sequences 5' → 3'
CMD26	Forward	In *glmS* for screening integration in *att*Tn7 site (used with CMD1416)	GAT CTT CTA CAC CGT TCC GC
CMD1416	Reverse	In Tn7 (Used with CMD26)	GCT TTT TCA CAGCAT AAC TGG A
CMD2337	Forward	Amplification of the Km^r cassette from pKD4 vector (used with CMD2338)	TTTTTGTCAAGCGAAA GAGAGTAATCATTGTT TATTTAGCGTATTATC GAGTGTAGGCTGGAG CTGCTTC
CMD2338	Reverse		CTTTAAACAGAACCG GATAATCTAAAATAT GCCGCCCCAAAGGGC GGCATATGGGAATTA GCCATGGTCC
CMD2356	Forward	Screening for *ryfA* mutation (used with CMD2357)	TATTGGCATTGAAGCC GATG
CMD2357	Reverse		GATTTACCGGTTGAGC CAGA
CMD2362	Forward	Cloning of *ryfA* into pGP-Tn7-Cm plasmid and amplification of *ryfA* fragment from CFT073 for complementation (used with CMD2363)	GCAAGGCCTTCGCGA GGTACCAGCAGATTT ACCGGTTGAGC
CMD2363	Reverse		CGGGCTGCAGGAATT CCTCGAGCGTGACTTT AAACAGAACCG
CMD2364	Forward	Cloning of *ryfA* into pNM12 plasmid and amplification of *ryfA* fragment from CFT073 (used with CMD2365)	CGCAACTCTCTACTGT TTGGCCATCCGGCCCT TTCCGCCGTCT
CMD2365	Reverse		TCCCCGGGTACCATG GTGAATTCCCGAACA TATTGCGCCATTC
CMD2712	Forward	Inverse PCR from pIJ546 to generate pIJ588: pGP-Tn7-Cm::*ryfA* variant 1 (used with CMD2713)	GCACAATTGAAGATA CGGTGCTTTTGTATCG
2713	Reverse		GGTCCATGCGAAGAC CGCATC

CMD2714	Forward	Screening for point mutation in pIJ588 (used with CMD2721)	TCTTCGCATGGACCGC ACAATT
CMD2715	Forward	Inverse PCR from pIJ546 to generate pIJ589: pGP-Tn7-Cm::*ryfA* variant 2 (used with CMD2716)	TGCTAATGTATCGTAC TTATTGTTTCTGGTGC
CMD2716	Reverse		CCGTTACTTCATTGTG CGGTCCATGCG
CMD2717	Forward	Screening for point mutation in pIJ589 (used with CMD2721)	CGCACAATGAAGTAA CGGTGCTAATG
CMD2718	Forward	Inverse PCR from pIJ546 to generate pIJ590: pGP-Tn7-Cm::*ryfA* variant 3 (used with CMD2719)	TTTGTAACGTACTTAT TGTTTCTGGTGCG
CMD2719	Reverse		AAGCACCGTATCTTCA TTGTGCG
CMD2720	Forward	Screening for point mutation in pIJ590 (used with CMD2721)	AGATACGGTGCTTTTT GTAACG
CMD2721	Reverse		GCATTCGGGCTTACTT GCTGC
EM4969B	Forward	RNA probe antisense to the RyfA sRNA	CCTTTCCGCCGTCTCG CAAA
EM4970	Reverse		TAATACGACTCACTAT AGGGAGAAAAATATG CCGCCCCAAAGGG
**qRT-PCR**			
CMD392	Forward	*rpoD* amplification (used with CMD393)	GCGTGAAGCGAAAGT TCTGCGTAT
CMD393	Reverse		TCGCGGGTAACGTCG AACTGTTTA
CMD1267	Forward	*fimA* amplification (used with CMD1268)	ACCGTTCAGTTAGGA CAGGTTCGT
CMD1268	Reverse		CGAGAGCCAGAACGT TGGTAT
CMD2370	Forward	*ryfA* amplification (used with CMD2371)	GTTTCTGGTGCGCTGT TA
CMD2371	Reverse		GCTTACTTGCTGCTCT GAA
CMD2547	Forward	*ibpA* amplification (used with CMD2548)	CTGAGAGCGAACTGG AAATTA
CMD2548	Reverse		ATGCCCTGGTACAGA

			TAGG
CMD2549	Forward	*cadA* amplification (used with CMD2550)	ACCAACTTCTCACCGATTTAC
CMD2550	Reverse		CCAGCAGTTTGTGAGTAGAC
CMD2557	Forward	*treC* amplification (used with CMD2558)	ACGTGGAGAGCCTCAATA
CMD2558	Reverse		GACTGTTGTCACGGGATTT
CMD2561	Forward	*cspA* amplification (used with CMD2562)	AAAGGCTTCGGCTTCATC
CMD2562	Reverse		GACCTTCGTCCAGAGATTTG
CMD2563	Forward	*soxS* amplification (used with CMD2564)	TTATCGCATGGATTGACGAG
CMD2564	Reverse		GGAACATCCGTTGCAAGT
CMD2575	Forward	*marA* amplification (used with CMD2576)	CTTCGAGTCCCAACAAACTC
CMD2576	Reverse		GCGATTCACCCTGCATATT
CMD2577	Forward	*bssS* amplification (used with CMD2578)	AATAAGTCCGAGCAGGAAGG
CMD2578	Reverse		TGGCGATTCCTGCTTCTAATA
CMD2605	Forward	*rpoH* amplification (used with CMD2606)	AACATCGGCCTGATGAAAG
CMD2606	Reverse		ATTCGTGGATCTCTGCTTTG
